# Activation of TFEB-mediated autophagy by trehalose attenuates mitochondrial dysfunction in cisplatin-induced acute kidney injury

**DOI:** 10.7150/thno.44051

**Published:** 2020-04-27

**Authors:** Lingling Zhu, Yujia Yuan, Longhui Yuan, Lan Li, Fei Liu, Jingping Liu, Younan Chen, Yanrong Lu, Jingqiu Cheng

**Affiliations:** NHC Key Laboratory of Transplant Engineering and Immunology, Department of Nephrology, Regenerative Medicine Research Center, West China Hospital, Sichuan University, Chengdu, China

**Keywords:** autophagy, transcription factor EB, mitochondrial dysfunction, acute kidney injury, trehalose

## Abstract

**Aims:** Cisplatin, an anticancer drug, always leads to nephrotoxicity by causing mitochondrial dysfunction. As a major mechanism for cellular self-degradation, autophagy has been proven to protect against cisplatin-induced acute kidney injury (AKI). Based on the activation of autophagy induced by trehalose, we aimed to investigate the nephroprotective effects of trehalose on cisplatin-induced AKI and its underlying mechanisms.

**Results:** Due to the activation of autophagy, mitochondrial dysfunction (mitochondrial fragmentation, depolarization, reactive oxygen species (ROS), and reduced ATP generation) and apoptosis induced by cisplatin were markedly inhibited in trehalose-treated HK2 cells in vitro. Based on the transcriptional regulation role of transcription factor EB (TFEB) in autophagy and lysosome, we characterized trehalose-induced nuclear translocation of TFEB. Furthermore, consistent with trehalose treatment, overexpression of TFEB inhibited cell injury induced by cisplatin. However, the protective effects of trehalose were largely abrogated in tfeb-knockdown cells. In vivo, cisplatin injection resulted in severe kidney dysfunction and histological damage in mice. Trehalose administration activated TFEB-mediated autophagy, alleviated mitochondrial dysfunction and kidney injury in AKI mice.

**Innovation and conclusion**: Our data suggest that trehalose treatment preserves mitochondria function via activation of TFEB-mediated autophagy and attenuates cisplatin-induced kidney injury.

## Introduction

Acute kidney injury (AKI), characterized by sudden renal function decline, is a worldwide public health problem associated with increased morbidity and mortality 1. Cisplatin, one of the first generation chemotherapeutic drugs, is a potent chemotherapeutic agent used for treating many kinds of solid tumors 2. However, its clinical use is limited by its side effects to normal tissues 3, 4. It has been reported that approximately 25% - 30% of patients treated with cisplatin might develop nephrotoxicity, such as AKI 5. Thus, challenges in the prevention of cisplatin nephrotoxicity still lie ahead.

Recent publications suggest that cisplatin induces tubular cell damage via multiple signaling pathways and factors 6. Among these, mitochondria are an important target organelle in cisplatin-induced kidney injury 7, 8. Due to accumulation in the mitochondria of renal proximal tubular cells, cisplatin evokes the subsequent excessive production of reactive oxygen species (ROS), decreasing membrane potential and impairing the mitochondrial redox balance, leading to cisplatin-induced renal injury 9-11.

Autophagy is a self-degradation process through which cells remove misfolded proteins, defective organelles, lipid droplets, and damaged DNA to maintain cellular homeostasis 12. Recent studies have demonstrated that basal autophagy in the kidney is vital for normal homeostasis of the proximal tubules, and downregulation of autophagy is associated with severe ischemia-reperfusion-induced acute kidney injury 13. Moreover, autophagy deficiency in the proximal tubule with conditional autophagy-related genes worsened tubular injury and renal function by aggravating mitochondrial damage in the AKI model 14, 15. Conversely, several strategies for enhancing autophagy could protect renal tubular cells from many stressors 16, 17. Thus, selectively targeting autophagy might be effective to alleviate kidney injury in AKI models. More importantly, Lynch/Parikh have found that the PGC1α-dependent renal stress resistance in cisplatin-induced AKI mice was relied on TFEB-driven lysosomal biogenesis and mitophagy, suggesting that TFEB may be a potentially novel target for renal tubular stress resistance 18.

Trehalose, a natural nonreducing disaccharide that is present in a diverse range of organisms, including bacteria, yeast, fungi, and plants, is a novel autophagy and TFEB inducer in many cells through an mTOR-independent pathway 19. Indeed, a recent study has reported that trehalose prevents osteoarthritis development, and administration of trehalose to osteoarthritis mice ameliorates oxidative stress-mediated mitochondrial dysfunction and ER stress via selective autophagy stimulation and autophagic flux restoration 20. In addition, Evans et al. found that trehalose could drive the macrophage autophagy-lysosome system to protect atherosclerosis by activating TFEB 21. However, the effects of trehalose on kidney injury in AKI mice has never been tested.

The aim of this study was to test whether trehalose administration could reduce renal dysfunction in cisplatin-induced AKI mice and, if so, whether these effects were relied on the activation of TFEB-mediated autophagy.

## Materials and Methods

### Materials and reagents

Cisplatin, hydroxychloroquine (HCQ), and rapamycin (Rapa) were purchased from MedChemExpress (MCE, New Jersey, USA). Trehalose was obtained from ApexBio (Boston, MA, USA). The cell counting kit 8 (CCK8) assay was purchased from Dojindo (Kumamoto, Japan). An Annexin V/PI apoptosis detection kit was purchased from BD Corporation (San Jose, CA, USA). MitoSOX Red, Mito deep Red and Hoechst were obtained from Thermo Fisher Scientific (Sunnyvale, CA, USA). A mitochondrial membrane potential (MMP) assay kit (JC-1) was purchased from AAT Bioquest (Sunnyvale, CA). Mito Tracker Green, an ATP measurement kit, and a nuclear and cytoplasmic protein extraction kit were purchased from Beyotime Biotechnology (Jiangsu, China). The tfeb-Fag-His plasmid (CH898579) was purchased from Vigene Biosciences (Shandong, China). The tfeb-siRNA was synthesized by GenePharma (Shanghai, China). The jetPRIME transfection reagent was obtained from Polyplus Transfection (Illkirch, France). The reagents used for transmission electron microscopy (osmium tetroxide, Epon, and lead citrate) and DAPI were purchased from Sigma-Aldrich (Taufkirchen, Germany). The primary antibodies were purchased from the following sources: anti-Bax, anti-LC3B, anti-Drp1, anti-Sirt3, and anti-TFEB (Cell Signaling Technology, Beverly, MA); anti-Bcl-2, anti-Parkin, anti-OPA1, anti-ATP5a, and anti-Ndufs4 (ABclonal Biotech Co., Ltd., Cambridge, MA, USA); anti-P62 (Abcam); and anti-Pink1 (Hua Bio-Engineering Co. Ltd., Shanghai, China). For real-time PCR, TRIzol was purchased from Gibco (Life Technologies, CA, USA), the iScript cDNA synthesis kit was obtained from Bio-Rad (CA, USA), and the SYBR Green PCR mix was purchased from Vazyme Biotech (Nanjing, China).

### Cell culture and treatment

The human renal proximal tubular cells (HK2) cell line was obtained from the American Type Culture Collection (ATCC) and cultured in DMEM/F-12 medium (HyClone, Solarbio, Beijing, China) supplemented with 10% fetal bovine serum, penicillin (100 U/mL) and streptomycin (100 μg/mL) and maintained at 37 °C in 5% CO_2_ in a humidified incubator.

Cells incubated with 5 µM cisplatin for 48 h were treated with or without trehalose. To induce or inhibit autophagy, cells were cocultured with Rapa (50 nM) or HCQ (20 μM) at the same time.

### Cell transfection

Small interfering RNA against tfeb (si*tfeb*) (sense: GACGAAGGUUCAACAUCAATT; antisense: UUGAUGUUGAACCUUCGUCTT) or negative control RNAi (sicon) were purchased from GenePharma (Shanghai, China). The tfeb plasmid with C-terminal flag and His tag (CH898579) was purchased from Vigene Biosciences (Shandong, China). To induce the overexpression or knockdown of TFEB, HK2 cells were transfected with tfeb-Fag-His plasmid and si*tfeb* using the jetPRIME transfection reagent.

### Cell viability and apoptosis assay

Cell viability was determined by a CCK8 assay kit. Briefly, 10 µl of CCK8 solution was added to each well containing 100 µl of medium. After incubating for 2 h, the absorbance was detected at 450 nm. Cell apoptosis was determined using an Annexin V/PI Apoptosis Detection kit following the manufacturer's instructions. Cells were incubated with Annexin V-FITC and/or propidium iodide (PI) for 30 min in the dark, and then apoptotic cells were analyzed via flow cytometry (Beckman, USA).

### Isolation of nuclear and cytoplasmic proteins and western blot analysis

Nuclear and cytoplasmic lysates were obtained using the Nuclear and Cytoplasmic Protein Extraction kit. For western blotting, tissue samples or cells were extracted using RIPA lysate containing protease inhibitor cocktail, and an immunoblot assay of the proteins was performed as described previously 22. Densitometry analysis was performed using ImageJ software. The relative fold differences in expression levels were normalized to the β-actin levels.

### Immunofluorescence

For the imaging of mitophagy, cells were incubated with 100 nM MitoTracker Deep Red at 37 °C for 30 min, fixed with 4% paraformaldehyde, permeabilized with 0.1% Triton X-100 for 10 min, and blocked in 1% BSA in PBS for 30 min. Then, the cells were immunolabeled with primary antibodies (LC3B at a 1:200 ratio; TFEB at a 1:100 ratio) overnight at 4 °C. After washing, the cells were incubated with a corresponding FITC-conjugated secondary antibody (1:200) in 1% BSA for 1 h at 37 °C. Nuclei were stained with DAPI for 5 min at room temperature. The fluorescent signals were examined using a fluorescence microscope (Zeiss, Germany).

### Mitochondrial morphology, mitochondrial ROS and mitochondrial membrane potential (Δψm) assessment

Cells were seeded and grown on glass coverslips. After incubating the cells with MitoTracker Green at 37 ℃ for 30 min, the mitochondrial morphology was visualized, and images were acquired using a confocal laser scanning microscope with 63× oil immersion objective lens. Mitochondrial ROS generation was evaluated by MitoSOX Red (2.5 μM) for 30 min at 37 ℃ and then analyzed by flow cytometry. The Δψm was evaluated by JC-1 (5 nM) for 30 min at 37 °C and then visualized, images were acquired using confocal microscopy (Nikon, Japan). The Δψm were analyzed by ImageJ, and the values are expressed as the fold-increase in red/green fluorescence over control cells.

### ATP measurement

ATP levels were measured using the ATP assay kit according to the manufacturer's instructions. Briefly, the collected cells and tissues were lysed with lysis buffer and then centrifuged at 12000 g for 10 min at 4 °C. After that, an aliquot of the supernatant plus ATP detection solution was added to a 96-well plate. Luminescence was detected using a SpectraMax M5 MultiMode Microplate Reader, and the ATP level is presented as nmol/mg of protein.

### Real-time PCR quantification

Total RNA was extracted by TRIzol and reverse-transcribed into cDNA with an iScript cDNA synthesis kit. Real-time polymerase chain reaction (real-time PCR) was performed using SYBR Green PCR mix (Vazyme Biotech) in a real-time PCR detector (Bio-Rad). The primer sequences used are listed in Table 1. Data analysis was performed using the ΔΔCt method.

### Animal experiments

Male C57BL/6 mice (6 - 8 weeks) were purchased from Dashuo Biotechnology (Chengdu, China), and all mice were housed in the animal center of West China Hospital, Sichuan University in accordance with the Guide for the Care and Use of Laboratory Animals. To evaluate the effects of trehalose on cisplatin-induced acute injury, mice were assigned to 3 groups (normal control, NC: n ≥ 8; cisplatin-treated, Cisp: n ≥ 8; cisplatin + trehalose, Cisp + Tre: n ≥ 8). Specifically, mice were intraperitoneally (i.p.) injected with cisplatin (16 mg/kg, single injection) and then administered trehalose (3 g/kg per day, i.p. or i.g.) for 2 days. The blood and kidneys were collected when the animals were sacrificed.

### Serum biochemistry

Plasma samples were separated by centrifugation at 1000 × g for 15 min. Clinical biochemistry analysis of creatinine (Crea) and urea nitrogen (BUN) from mice were measured by the Department of Laboratory Medicine of West China Hospital (Chengdu, China).

### Terminal deoxynucleotidyl transferase-mediated dUTP nick-end labeling (TUNEL) staining

Apoptotic cell death in kidney sections was determined using TUNEL staining (Promega, Madison, WI, USA). Briefly, kidney sections were deparaffinized and pretreated with 0.1 M sodium citrate, pH 6.0, at 65 °C for 30 min and then incubated with a TUNEL reaction mixture for 1 h at 37 °C in a dark chamber, and the nuclei were labeled by DAPI. Positive staining with nuclear DNA fragmentation was detected by fluorescence microscopy (Zeiss, Germany).

### Histological assessment

The kidney tissues were dissected and fixed in 10% formalin. Fixed tissues were processed for embedding in paraffin, and 5 µm sections were prepared, stained with hematoxylin-eosin (HE) and periodic acid Schiff (PAS), and observed by light microscopy (Zeiss, Germany).

### Transmission electron microscopy (TEM)

Kidney tissues were fixed in paraformaldehyde and glutaraldehyde, postfixed in osmium tetroxide, dehydrated in ethanol, subjected to resin penetration and embedded in Epon. After that, the tissues were cut into ultrathin sections and stained with uranyl acetate, followed by staining with lead citrate, and examined with a FEI Tecnai Spirit (T12). The aspect ratio and mitochondrial area were calculated.

### Isolation of mouse primary tubule epithelial cells (mPRTCs)

Isolation of mPRTCs was performed under a modified protocol as described previously 23, 24. Briefly, kidney cortices were minced with a sterile scalpel, transferred to a collagenase solution (DMEM/F-12 medium with 0.1% (wt/vol) type IV collagenase and 0.1% bovine serum albumin) at 37 °C and digested for 30 min. After digestion, the supernatant was sieved through two nylon sieves (pore sizes 100 μm and 40 μm). Then, the proximal tubule (PT) fragments remaining in the 40 μm sieve were resuspended by flushing the sieve in the reverse direction with warm media. The PTs present in the media were centrifuged for 5 min at 170 g, washed, resuspended in DMEM/F-12 medium supplemented with 10% fetal bovine serum and maintained at 37 °C in 5% CO2 in a humidified incubator.

### Statistical analysis

All data are expressed as the mean ± s.d. from at least three independent experiments.

Mann-Whitney analysis was employed for comparisons between two groups; one-way ANOVA (followed by Tukey's or LSD multiple comparison post hoc test) was used for groups of three or more. A value of P < 0.05 was considered statistically significant.

## Results

### Trehalose suppresses cisplatin-induced injury in vitro

**Figure 1:**
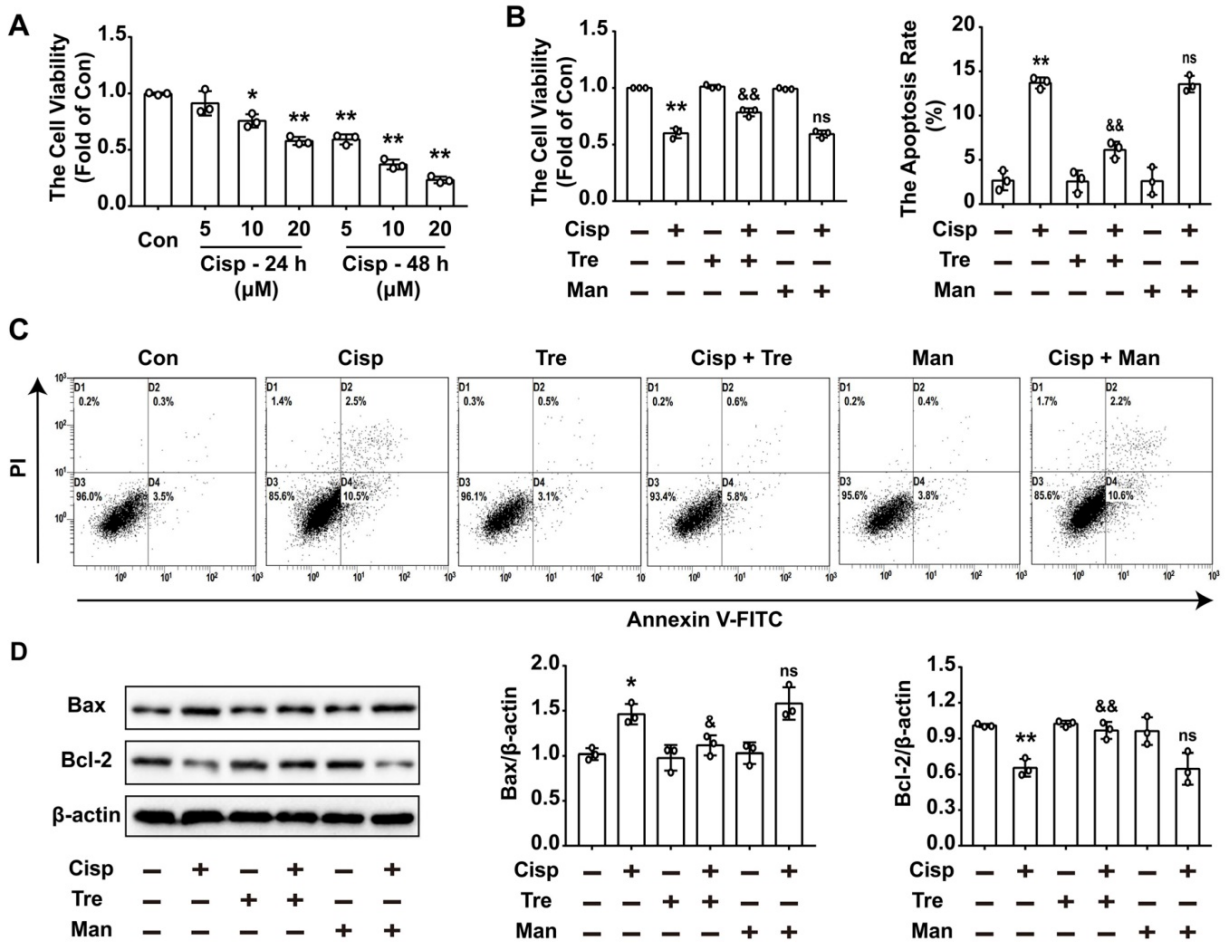
** Trehalose suppresses cisplatin-induced injury in vitro.** Mannitol was used as osmotic control of trehalose. (A) HK2 cells were exposed to cisplatin (5 μM, 10 μM, 20 μM) for 24 h or 48 h, and cell viability was determined by CCK8. (B) HK2 cells treated with cisplatin were incubated with trehalose for 48 h, and cell viability was determined. (C) The effects of trehalose on cisplatin-induced apoptosis were determined by flow cytometry. (D) The expression of apoptosis-related proteins (Bax and Bcl-2 was measured by western blotting. B - D, cisplatin, 5 μM; trehalose, 50 mM; mannitol, 50 mM. Data are shown as the means ± s.d. from three independent experiments and analyzed by one-way ANOVA with Tukey's test. * P < 0.05, ** P < 0.01 vs. Con; & P < 0.05, && P < 0.01 vs. Cisp. (Con, control; Tre, trehalose; Cisp, cisplatin; Cisp + Tre, cisplatin + trehalose; Man, mannitol; Cisp + Man, cisplatin + mannitol).

To evaluate the effects of trehalose in cisplatin-treated HK2 cells, cisplatin-treated HK2 cells were incubated with or without trehalose. Here, we found that after cisplatin treatment, cell viability was declined in a concentration- and time-dependent manner (Figure 1A). After incubation with trehalose, the cell viability was increased to 0.78 ± 0.04-fold from 0.60 ± 0.04-fold in cisplatin-treated HK2 cells (Figure 1B). In addition, cell apoptosis induced by cisplatin was significantly inhibited after trehalose treatment (Figure 1C). In parallel to ameliorating cell apoptosis, the enhanced expression of Bax was decreased after trehalose treatment, while the reduced expression of Bcl-2 was increased (Figure 1D). Mannitol was used as osmotic control of trehalose, and no significant effects were observed in the above parameters in osmotic control group. These data showed that trehalose protected against cisplatin-induced renal tubular cell apoptosis *in vitro*.

### Trehalose induces autophagy in cisplatin-treated HK2 cells

**Figure 2:**
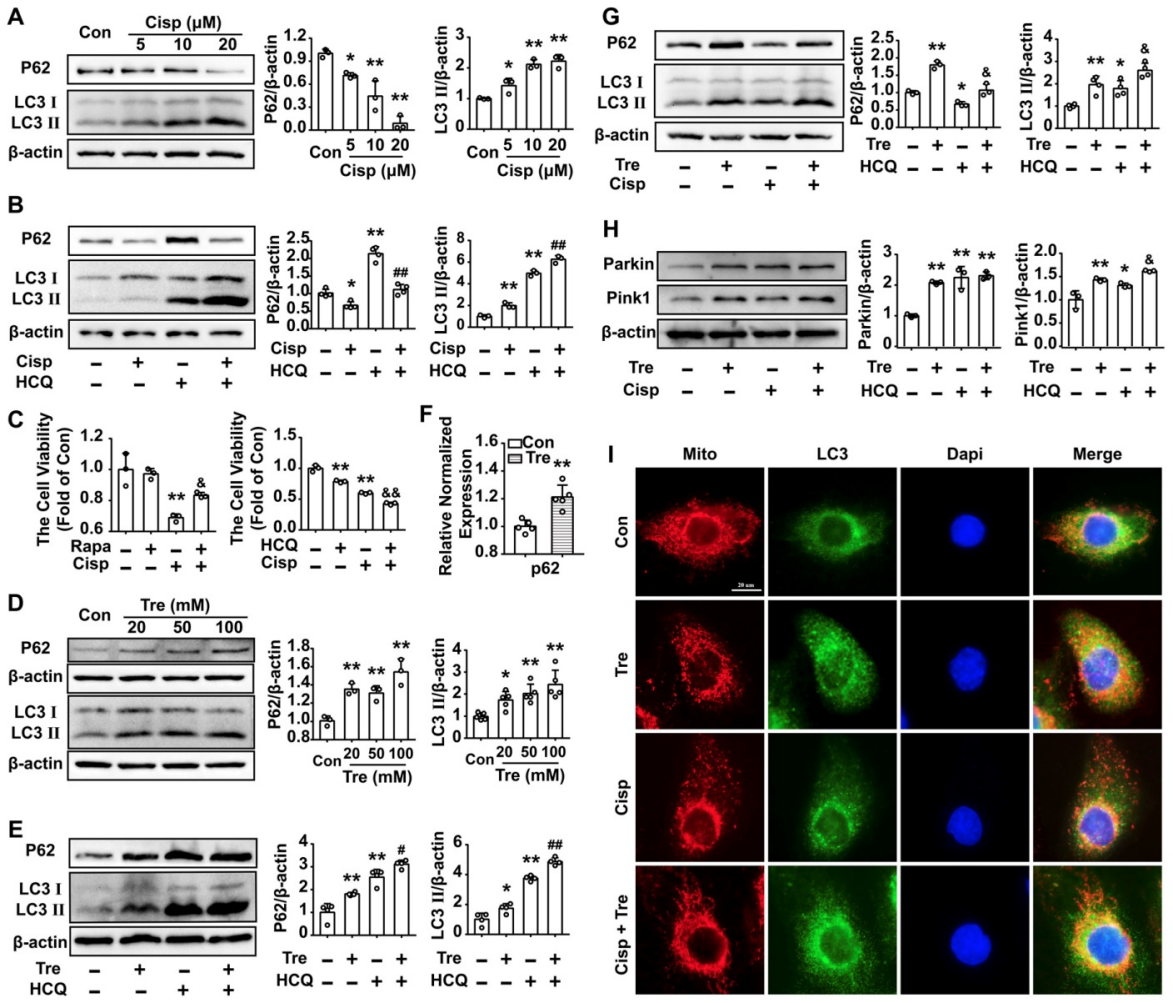
** Trehalose induces autophagy in cisplatin-treated HK2 cells.** (A) The expression of the proteins P62 and LC3 was measured by western blotting in HK2 cells exposed to cisplatin (5 μM, 10 μM, 20 μM). (B) The expression of the proteins P62 and LC3 was measured by western blotting in HK2 cells exposed to cisplatin (5 μM) in the presence or absence of HCQ (20 μM). (C) HK2 cells were treated with cisplatin (5 μM) in the presence or absence of Rapa (50 nM) or HCQ (20 μM), and cell viability was determined. (D) The expression of the proteins P62 and LC3 was measured by western blotting in HK2 cells exposed to trehalose (20 mM, 50 mM, 100 mM). (E) The expression of the proteins P62 and LC3 was measured by western blotting in HK2 cells exposed to trehalose (50 mM) in the presence or absence of HCQ (20 μM). (F) HK2 cells treated with trehalose (50 mM) and p62 mRNA was measured by real-time polymerase chain reaction (real-time PCR). Data analyzed by Mann-Whitney. (G and H) HK2 cells were exposed to cisplatin (5 μM) in the presence or absence of trehalose (50 mM), and the expression of the proteins P62, LC3, Pink1 and Parkin was measured by western blotting. (I) Representative images of the colocalization between LC3 and mitochondria. Data are shown as the means ± s.d. from at least three independent experiments. A - C and D - I, Data analyzed by one-way ANOVA with Tukey's test. * P < 0.05, ** P < 0.01 vs. Con; & P < 0.05, && P < 0.01 vs. Cisp; # P < 0.05, ## P < 0.01 vs. HCQ. (Con, control; Cisp, cisplatin; HCQ, hydroxychloroquine; Rapa, Rapamycin; Tre, trehalose; Cisp + Tre, cisplatin + trehalose).

The LC3 II level was increased in HK2 cells treated with cisplatin for 48 h, while the P62 level was decreased (Figure 2A). According to the guidelines for the use and interpretation of assays for monitoring autophagy (3rd edition), the accumulation of LC3 Ⅱ could be due to either the induction of autophagy or the inhibition of autophagic degradation 25, 26. Moreover, in the presence of the lysosomal acidification inhibitor HCQ, the LC3 II protein was dramatically elevated in cisplatin-treated HK2 cells, indicating that increased LC3 II turnover and induction of autophagic flux were induced by cisplatin in HK2 cells (Figure 2B). To explore the effect of autophagy on cisplatin-induced cell injury, an autophagy activator (mTOR inhibitor, Rapamycin; Rapa) or inhibitor (HCQ) was added to the cells. Compared with the cisplatin group, we found that cell viability was increased after Rapa treatment, while furtherly decreased in the cisplatin + HCQ group (Figure 2C). The results demonstrated that autophagy plays a protective role in cisplatin-induced cell injury.

Western blot analysis showed that LC3 II and P62 increased in trehalose-treated HK2 cells in a dose-dependent manner (Figure 2D). The accumulation of P62 level was correlated with dysfunctional autophagy or transcriptional activation of p62. Herein, the elevated protein of P62 was due to the increase of the p62 mRNA after trehalose treatment, which was consistent with the observation in NSC34 and Hepa1-6 cells (Figure 2F) 27, 28. As osmotic control, mannitol had no significant effect on LC3 II and P62 levels in HK2 cells (Figure S1A). Moreover, in the presence of HCQ, the LC3 Ⅱ and P62 proteins were elevated in trehalose-treated HK2 cells, suggesting that trehalose could enhance the autophagy process (Figure 2E and G). Mitophagy, as a selective autophagy, is critical for mitochondrial maintenance. Here, we found that the expression of Pink1 and Parkin were increased after trehalose treatment (Figure 2H). Additionally, the colocalization between LC3 and mitochondria was increased (Figure 2I), indicating that the mitophagy process was activated under this stimulus.

### Trehalose attenuates mitochondrial dysfunction in cisplatin-treated HK2 cells

**Figure 3:**
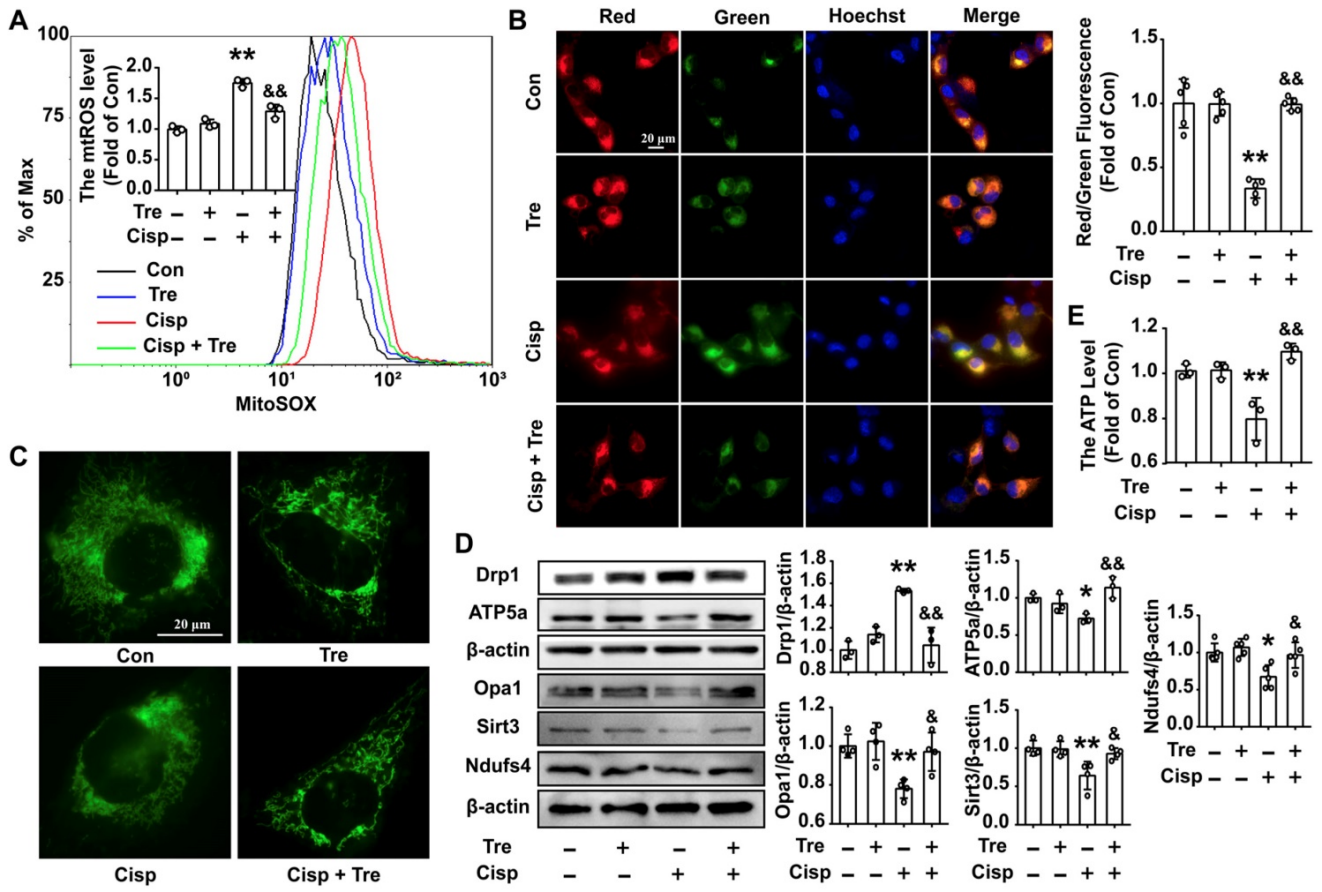
** Trehalose attenuates mitochondrial dysfunction in cisplatin-treated HK2 cells.** (A) Mitochondrial ROS (mtROS) were measured by incubation with MitoSOX. (B) The mitochondrial membrane potential measurement was detected with JC-1 and quantified by ImageJ. (C) Representative images of the mitochondrial morphology of HK2 cells stained with MitoTracker Green. (D) The expression of mitochondria-related proteins (Drp1, Opa1, ATP5a, Sirt3 and Ndufs4) was measured by western blotting. (E) ATP levels were quantified in HK2 cells, the ATP content was calculated as nmol/mg of protein, and the data are represented as the rate of control. (cisplatin, 5 μM; trehalose, 50 mM). Data are shown as the means ± s.d. from at least three independent experiments and analyzed by one-way ANOVA with Tukey's test. * P < 0.05, ** P < 0.01 vs. Con; & P < 0.05, && P < 0.01 vs. Cisp. (Con, control; Tre, trehalose; Cisp, cisplatin; Cisp + Tre, cisplatin + trehalose).

Due to the protective role of mitophagy on mitochondrial dysfunction, we then measured mitochondrial function in cisplatin-treated HK2 cells. After trehalose treatment, the overproduction of mitochondrial ROS (mtROS) and depolarization of membrane potential induced by cisplatin was inhibited (Figure 3A and B). Moreover, compared with normal cells, the mitochondria in cisplatin-treated HK2 cells were swollen and smaller, indicating that cisplatin induced mitochondrial fragmentation. Consistent with the alterations in mitochondrial morphology, the protein level of optic atrophy 1 (Opa1) was decreased, and the expression of the mitochondrial fission protein Drp1 was remarkably increased. While, trehalose treatment effectively alleviated mitochondrial fragmentation (Figure 3C and D). Parallel changes included increase of mitochondria-related proteins (ATP5a, Sirt3 and Ndufs4) were observed after trehalose treatment (Figure 3D). As a consequence, the ATP content in cisplatin-treated HK2 cells was increased when the cells were incubated with trehalose (Figure 3E). Mannitol, used as osmotic control, has no significant effects on overproduction of measured mtROS and decreased ATP level in cisplatin-induced HK2 cells (Figure S1B and C). Collectively, these results indicated that trehalose alleviated cisplatin-induced mitochondrial dysfunction by promoting autophagy.

### Trehalose induces autophagy by activating TFEB in HK2 cells

**Figure 4:**
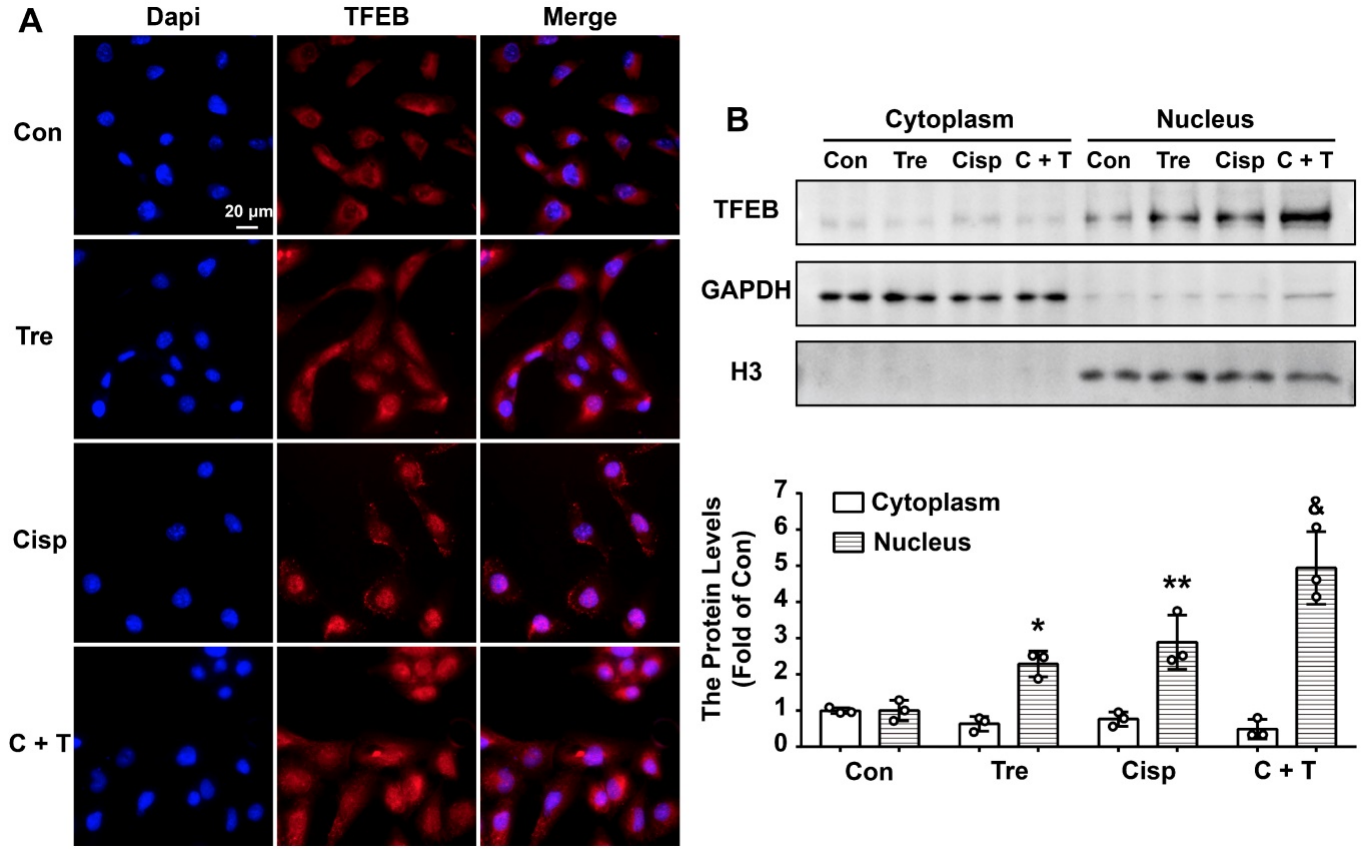
** Trehalose induces autophagy by activating TFEB in HK2 cells.** (A) Representative immunofluorescence images of TFEB in HK2 cells. (B) Cytoplasmic and nuclear fractions of TFEB in HK2 cells were analyzed by western blotting. (cisplatin, 5 μM; trehalose, 50 mM). Data are shown as the means ± s.d. from three independent experiments and analyzed by one-way ANOVA with Tukey's test. * P < 0.05, ** P < 0.01 vs. Con; & P < 0.05, && P < 0.01 vs Cisp. (Con, control; Tre, trehalose; Cisp, cisplatin; C + T, cisplatin + trehalose).

Although trehalose is one of the few known compounds to induce mTOR-independent autophagy effectively, its mechanism of action is completely unknown. More recently, transcription factor EB (TFEB), which is a major regulator of autophagy and lysosomal biogenesis, has emerged as a key factor in several disease pathologies 21. TFEB is predominantly diffusely located in the cytoplasm in the normal physiological state. Upon starvation stress, it translocates to the nucleus, resulting in activated transcription of its target genes. Based on these studies, we investigated whether TFEB is involved in the action of trehalose in HK2 cells. Confocal microscopy showed that under untreated conditions, endogenous TFEB was mainly distributed in the cell cytoplasm and progressively translocated into the nucleus after trehalose administration (Figure 4A). Similar results were also shown by western blotting of TFEB from the nucleic and cytosolic fractions of HK2 cells (Figure 4B). These data confirmed previous findings that trehalose could activate TFEB.

**Figure 5:**
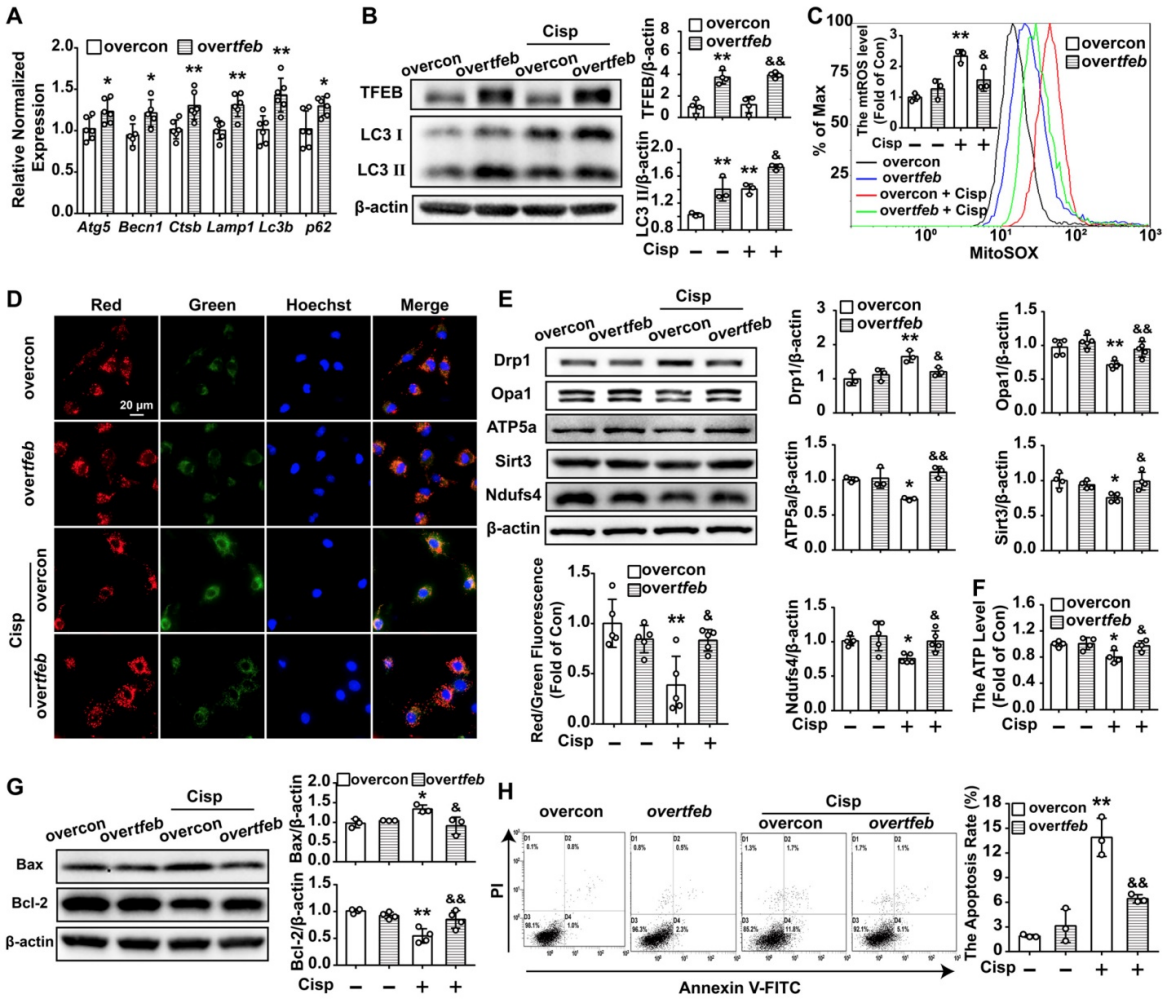
** Over-TFEB attenuates mitochondrial dysfunction and apoptosis in cisplatin-treated HK2 cells.** Control and TFEB-overexpressing HK2 cells treated or not with cisplatin (5 μM). (A) The mRNA levels of several autophagy-lysosome markers were evaluated by real-time PCR. Data analyzed by Mann-Whitney. (B) The protein levels of LC3 Ⅱ and TFEB were evaluated by western blotting. (C) mtROS were measured by incubation with Mito-SOX. (D) Representative images of JC-1 staining. (E) The expression of mitochondria-related proteins (Drp1, Opa1, ATP5a, Sirt3 and Ndufs4) was analyzed by western blotting. (F) ATP levels were assessed using an ATP assay kit. (G) The expression of the proteins Bax and Bcl-2 was measured by western blotting. (H) Apoptosis was determined by flow cytometry. Data are shown as the means ± s.d. from at least three independent experiments. B - H, Data analyzed by one-way ANOVA with Tukey's test. * P < 0.05, ** P < 0.01 vs. Con; & P < 0.05, && P < 0.01 vs. Cisp. (overcon, overcontrol; Cisp, cisplatin).

**Figure 6:**
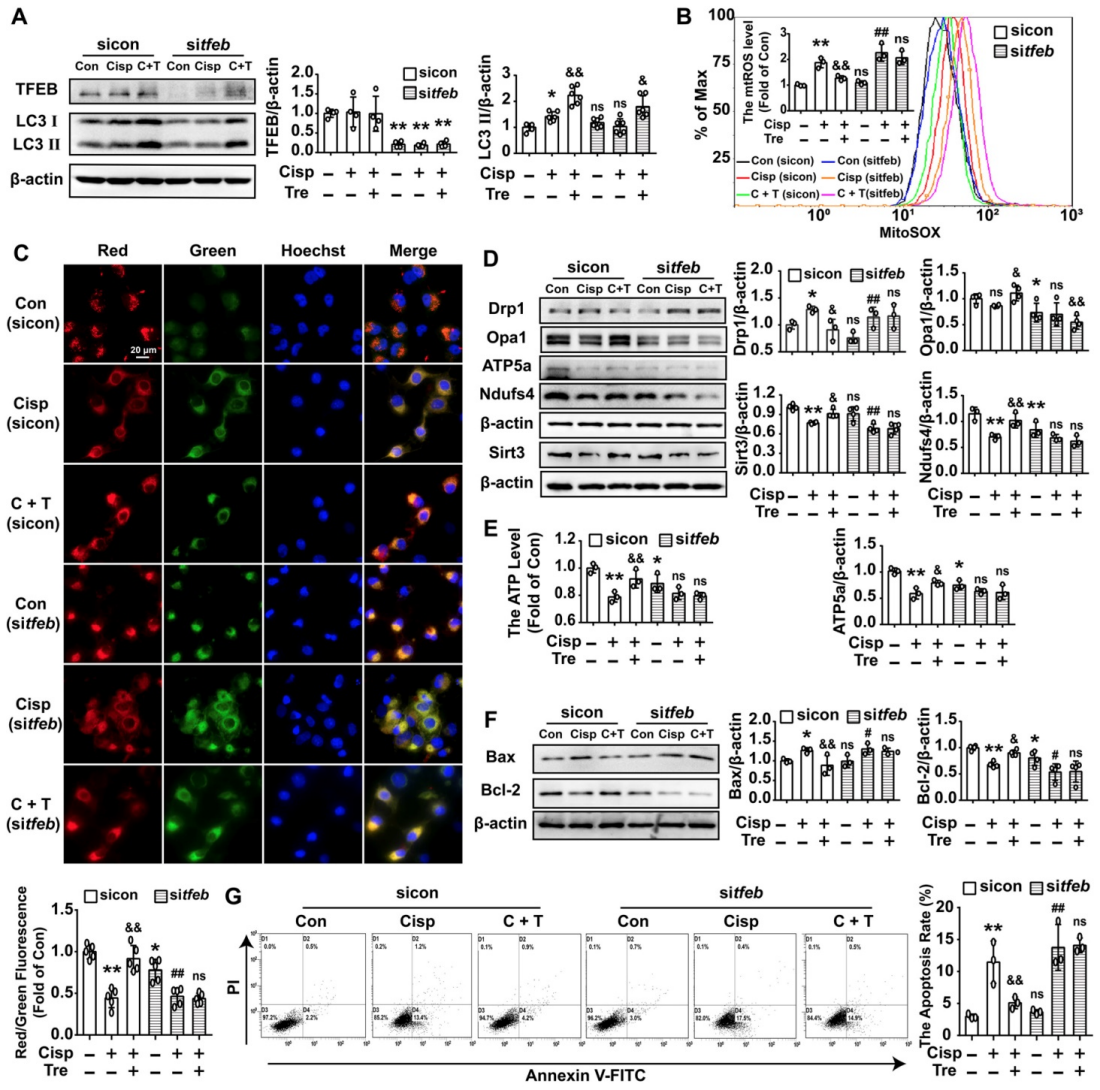
** Silencing of TFEB partially abolishes the protective effects of trehalose in cisplatin-treated HK2 cells.** HK2 cells were transfected with control siRNA (sicon) or TFEB siRNA (sitfeb) for 6 h and treated with cisplatin (5 μM) in the presence or absence of trehalose (50 mM) for 48 h. (A) The expression of autophagy-related proteins (TFEB and LC3) was measured by western blotting. (B) mtROS were measured by incubation with MitoSOX. (C) Representative images of JC-1 staining. (D) The expression of mitochondria-related proteins (Drp1, Opa1, ATP5a, Sirt3, and Ndufs4) was measured by western blotting. (E) ATP levels were assessed using an ATP Assay Kit. (F) The expression of apoptosis-related proteins (Bax and Bcl-2) was measured by western blotting. (G) The apoptosis of HK2 cells was determined by flow cytometry. Data are shown as the means ± s.d. from at least three independent experiments and analyzed by one-way ANOVA with LSD test. * P < 0.05, ** P < 0.01 vs. Con; & P < 0.05, && P < 0.01 vs. Cisp. # P < 0.05, ## P < 0.01 vs. sitfeb. (Con, control; Cisp, cisplatin; Tre, trehalose; C + T, cisplatin + trehalose).

To further determine whether the protective role of trehalose on cisplatin-induced injury was relied on TFEB-mediated autophagy, HK2 cells were transfected with the tfeb-Fag-His plasmid or tfeb-siRNA prior to trehalose treatment. Overexpression of TFEB could activate the transcription of autophagy- and lysosome-related genes, including *Atg5*, *Becn1*,* Ctsb*,* Lamp*, *Lc3b* and *p62* (Figure 5A). Moreover, along with the elevated expression of TFEB, the LC3 II level was significantly increased, indicating that overexpression of TFEB promoted the autophagy process in cisplatin-treated HK2 cells (Figure 5B). Furthermore, the impaired mitochondria induced by cisplatin were effectively improved in the over-*tfeb* group, characterized by decreased accumulation of mtROS, inhibition of depolarization of the membrane potential and mitochondrial fragmentation, and increased mitochondrial transport chain complex proteins (ATP5a and Ndufs4) and ATP content (Figure 5C-F). Finally, as a consequence, overexpression of TFEB increased cell viability and inhibited cell apoptosis induced by cisplatin treatment (Figure 5G and H). Conversely, the improvement of mitochondrial function and inhibition of cell injury after trehalose treatment was remarkably abrogated in tfeb-knockdown HK2 cells (Figure 6). These results indicated that upon trehalose treatment, TFEB was crucial for the induction of autophagy and protection against cell damage in cisplatin-treated HK2 cells.

### Intraperitoneal administration of trehalose prevents cisplatin-induced AKI

**Figure 7:**
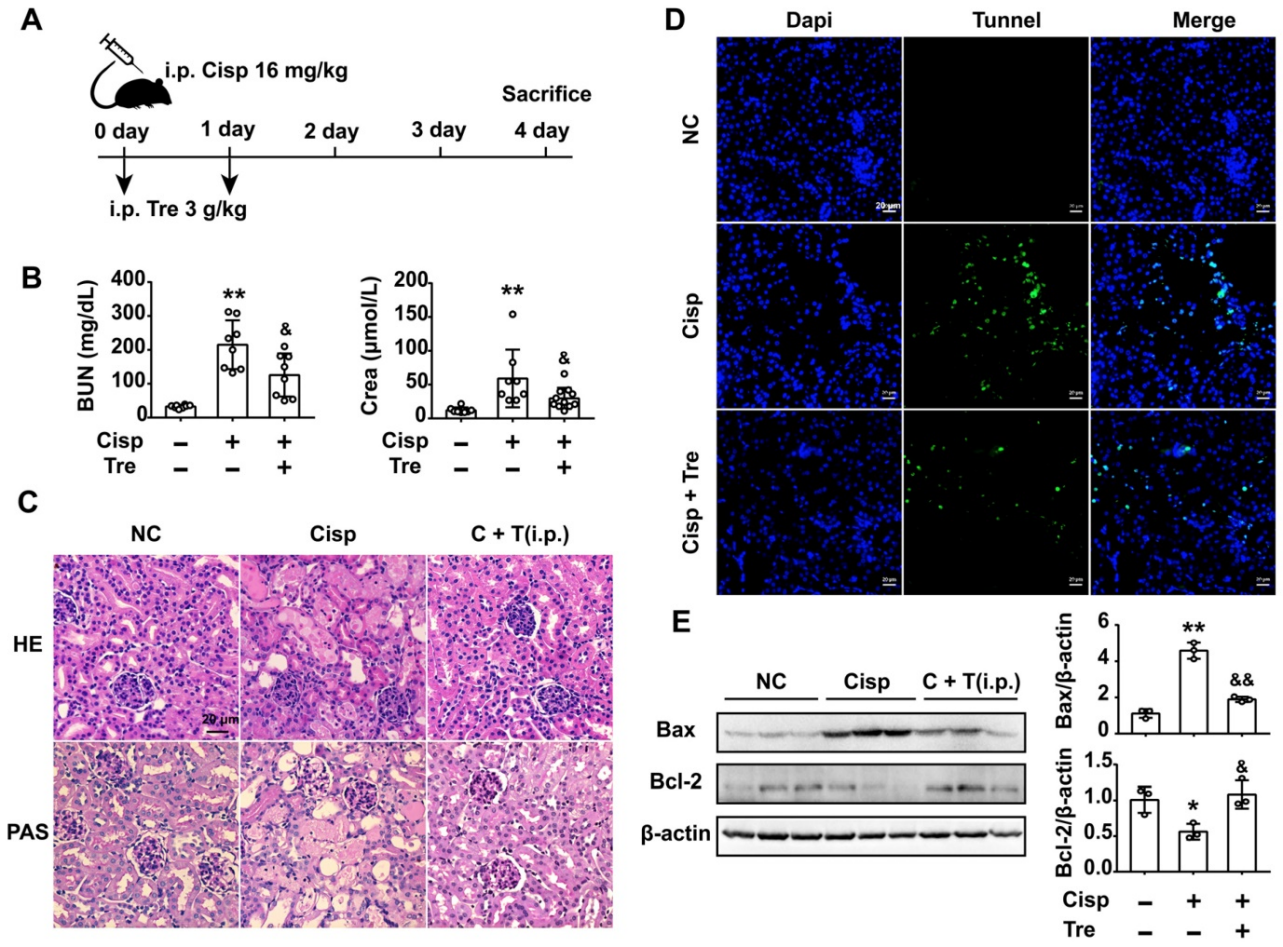
** Intraperitoneal (i.p.) injection of trehalose protects the kidney against cisplatin-induced AKI.** (A) Mice were intraperitoneally injected with a single dose of cisplatin (16 mg/kg BW) to induce AKI and then intraperitoneally administered trehalose (3 g/kg BW) at the same time for 2 days. (B) Serum BUN and Crea levels were quantified in each group (n = 8 - 11 mice). (C) Representative images of HE- and PAS-stained kidney sections. (D) Representative micrographs showing TUNEL staining. (E) Representative images of western blotting and the quantitative analyses of Bax and Bcl-2 (n = 3 mice). Data analyzed by one-way ANOVA with Tukey's test. * P < 0.05, ** P < 0.01 vs. Con; & P < 0.05, && P < 0.01 vs. Cisp. (NC, normal control; Cisp, cisplatin; Tre, trehalose; C + T, cisplatin + trehalose; i.p., intraperitoneal; BUN, blood urea nitrogen; Crea, serum creatinine; HE, hematoxylin/eosin; PAS, periodic acid-Schiff; TUNEL, terminal deoxynucleotidyl transferase-mediated dUTP nick-end labeling staining).

To evaluate the preventive effects of trehalose *in vivo*, male C57BL/6N mice were intraperitoneally injected with cisplatin (16 mg/kg) to induce AKI. For trehalose administration, AKI mice were intraperitoneally injected with trehalose (i.p., 3 g/kg, once a day for 2 days) at the beginning of AKI (Figure 7A). Here, we found that trehalose treatment for 4 days had no effects on mouse blood urea nitrogen (BUN) and serum creatinine (Crea) levels, suggesting that trehalose did not induce nephrotoxicity in mice (Figure S2). After cisplatin injection, the levels of BUN and Crea were increased on the 4th day, while decreased after trehalose administration (Figure 7B). In addition, histological staining of the kidney sections showed that cisplatin-treated mice displayed histopathological alterations, such as loss of the brush border, tubular cell loss, and cast formation, whereas renal structural damage was attenuated by trehalose administration (Figure 7C).

It is well known that cisplatin induces apoptosis of proximal tubular epithelial cells. We next assessed apoptosis in the kidneys, and apoptotic cells were detected by terminal deoxynucleotidyl transferase-mediated dUTP nick end labeling (TUNEL) staining. Here, we found that the number of TUNEL-positive cells was lower in the trehalose-treated group than in the cisplatin group (Figure 7D). Accordingly, significantly higher expression of the pro-apoptotic Bax treated with cisplatin was decreased and lower expression of anti-apoptotic Bcl-2 was increased after trehalose administration (Figure 7E). Taken together, these data indicated that trehalose had a protective effect on cisplatin-induced AKI.

### Intraperitoneal administration of trehalose activates autophagy and improves mitochondria in AKI mice

**Figure 8:**
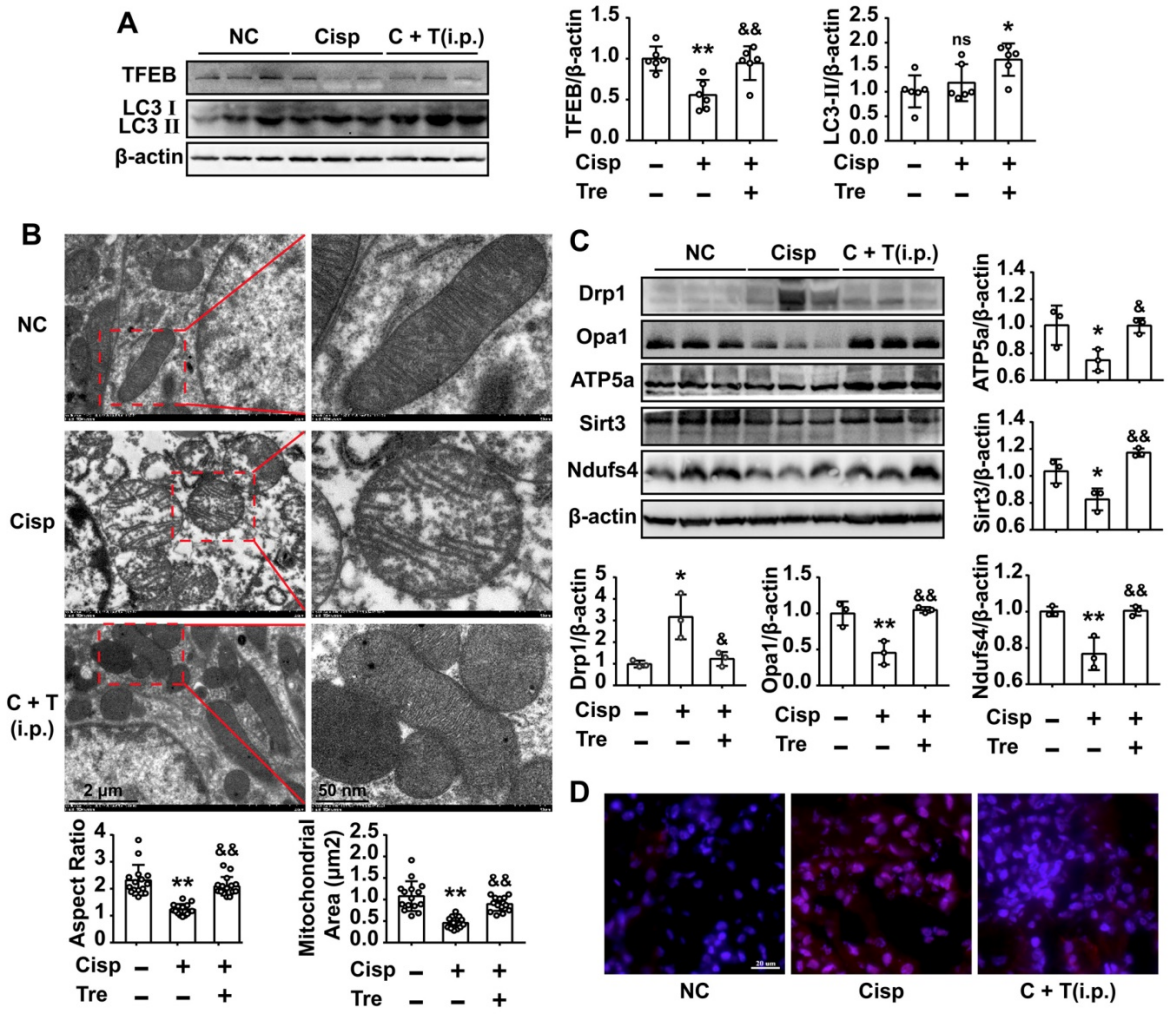
** Intraperitoneal injections of trehalose activate autophagy and improve mitochondria in AKI mice.** (A) The expression of autophagy-related proteins (TFEB and LC3) was measured by western blotting (n = 6 mice). (B) Representative TEM micrographs of mouse renal tubular epithelial cell mitochondria from each group. Scale bar, 2 μm (wireframe indicates the magnified image). The aspect ratio (major axis/minor axis) of mitochondria and the mitochondrial area were evaluated via ImageJ (n = 15 mitochondria). (C) The expression of mitochondria-related proteins (Drp1, Opa1, ATP5a, Sirt3 and Ndufs4) was measured by western blotting (n = 3 mice). (D) Representative images showing mtROS in fresh renal tissue visualized by staining with MitoSOX. Data analyzed by one-way ANOVA with Tukey's test. * P < 0.05, ** P < 0.01 vs. Con; & P < 0.05, && P < 0.01 vs. Cisp. (NC, normal control; Cisp, cisplatin; C + T, cisplatin + trehalose; i.p., intraperitoneal).

As shown in the previous study, Lynch/Parikh reported that at the early stage of cisplatin-induced AKI the mitophagy was induced as an adaptive response, while was retarded along with the development of AKI, thereby accelerating cell death 18. Consistent with the report, we found that the mRNA levels of autophagy related genes (*Becn1*, *Ctsb*, *Lc3b*) was elevated at 2d, and sequentially reduced at 4d after cisplatin injection (Figure S3B). Meanwhile, the protein level of LC3 II was slightly elevated without significant difference between NC and AKI mice, and the TFEB level was decreased in AKI mice (Figure 8A). Our data showed that cisplatin induced autophagy and the process may be retarded by a later time.

Moreover, western blot analysis showed that the expression of LC3 II in the kidney sections was increased after trehalose administration, indicating that trehalose activated autophagy in AKI mice (Figure 8A). Based on the protective role of autophagy on mitochondrial function, we observed the mitochondria in the cortices of the mouse kidneys using transmission electron microscopy. Compared with the normal control (NC) group, many swollen, smaller and round mitochondria were observed in the tubular cells of AKI mice, and quantitative morphometric analysis revealed that the aspect ratio (the long axis of mitochondria/short axis of mitochondria) and mitochondrial area were decreased, which is indicative of mitochondrial fragmentation. However, these phenomena were obviously attenuated after trehalose administration (Figure 8B). In line with the alterations in mitochondrial morphology, the elevated expression of Drp1 in AKI mice was decreased in the trehalose-administrated group (Figure 8C). In addition, the expression levels of mitochondrial transport chain complex proteins (ATP5a and Ndufs4) in the kidney were increased in the trehalose-administrated group (Figure 8C). Moreover, compared with the NC mice, the mtROS levels in the kidney, as assessed by MitoSOX fluorescence, was increased in cisplatin-induced AKI mice, while markedly decreased after trehalose administration (Figure 8D).

Due to the accumulation of cisplatin in the mitochondria of renal proximal tubular cells *in vivo*, we then isolated mouse primary tubule epithelial cells (mPRTCs) and measured their mitochondrial function. Our results showed that trehalose promoted TFEB translocation to nuclear and the autophagy process was promoted in the mPRTCs from trehalose-administrated group mice (increased expression of LC3 II) (Figure S4A and B). Along with the activation of autophagy, mitochondrial dysfunction in mPRTCs from AKI mice were alleviated, such as decreased mtROS levels, an elevated membrane potential, and increased ATP content (Figure S4C-F). In addition, the cisplatin-induced apoptosis in mPRTCs from AKI mice, was inhibited after trehalose administration (Figure S4G and H). Collectively, these results indicated that the beneficial effects of trehalose on cisplatin-induced acute kidney injury were probably due to the prevention of mitochondrial dysfunction.

### Oral administration of trehalose prevents cisplatin-induced AKI

**Figure 9:**
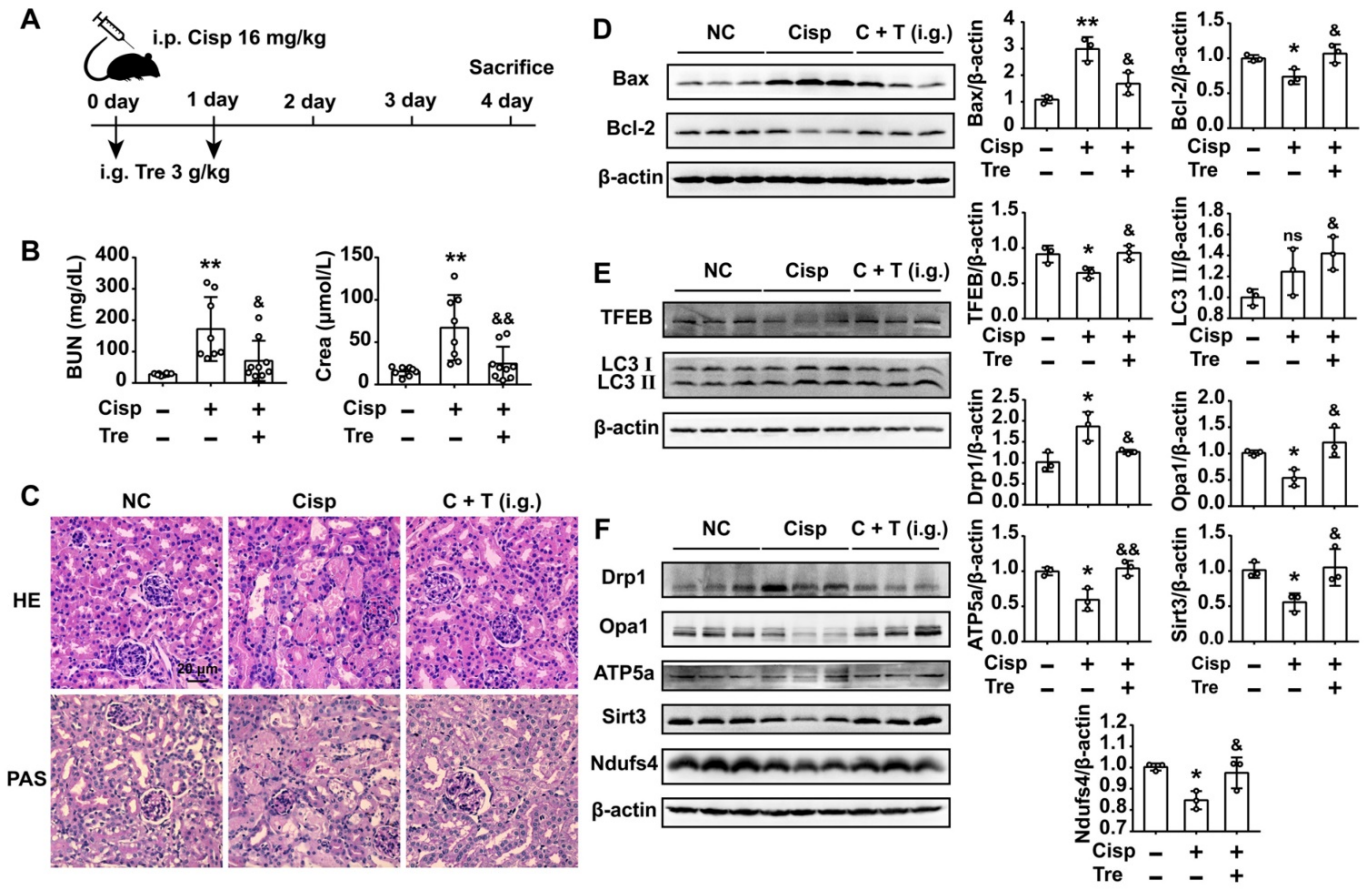
** Oral administration of trehalose prevents cisplatin-induced AKI.** (A) Mice intraperitoneally injected with cisplatin were orally administered trehalose at the same time for 2 days. (B) Serum BUN and Crea levels were quantified in each group (n = 8 - 10 mice). (C) Representative images of HE- and PAS-stained kidney sections. (D - F) The expression levels of apoptosis-related proteins (Bax and Bcl-2), autophagy-related proteins (TFEB and LC3), and mitochondria-related proteins (Drp1, Opa1, ATP5a, Sirt3 and Ndufs4) were measured by western blotting (n = 3 mice). Data analyzed by one-way ANOVA with Tukey's test. * P < 0.05, ** P < 0.01 vs. Con; & P < 0.05, && P < 0.01 vs. Cisp. (NC, normal control; Cisp, cisplatin; Tre, trehalose; C + T, cisplatin + trehalose; i.g., intragastric; BUN, blood urea nitrogen; Crea, serum creatinine; HE, hematoxylin/eosin; PAS, periodic acid-Schiff).

For the consideration of trehalose in clinical therapeutic usage, we furtherly tested whether oral administration of trehalose could alleviate kidney injury in AKI mice. Male C57BL/6N mice were intraperitoneally injected with cisplatin and orally administered trehalose at the same time (Figure 9A). Compared with AKI mice, kidney injury was alleviated after the oral administration of trehalose, as indicated by decreased levels of BUN and Crea, and decreased pathological damage (Figure 9B and C). Moreover, oral administration of trehalose decreased the level of pro-apoptotic Bax and increased anti-apoptotic Bcl-2 expression in AKI mice (Figure 9D). In terms of autophagy, we found that the expression levels of TFEB and LC3 II were increased, indicating that oral administration of trehalose activated autophagy in AKI mice (Figure 9E). As a consequence of activated autophagy, the impaired mitochondria (mitochondrial fragmentation and decreased expression of mitochondrial transport chain complex proteins) was improved (Figure 9F).

### Oral treatment with trehalose alleviates cisplatin-induced AKI

**Figure 10:**
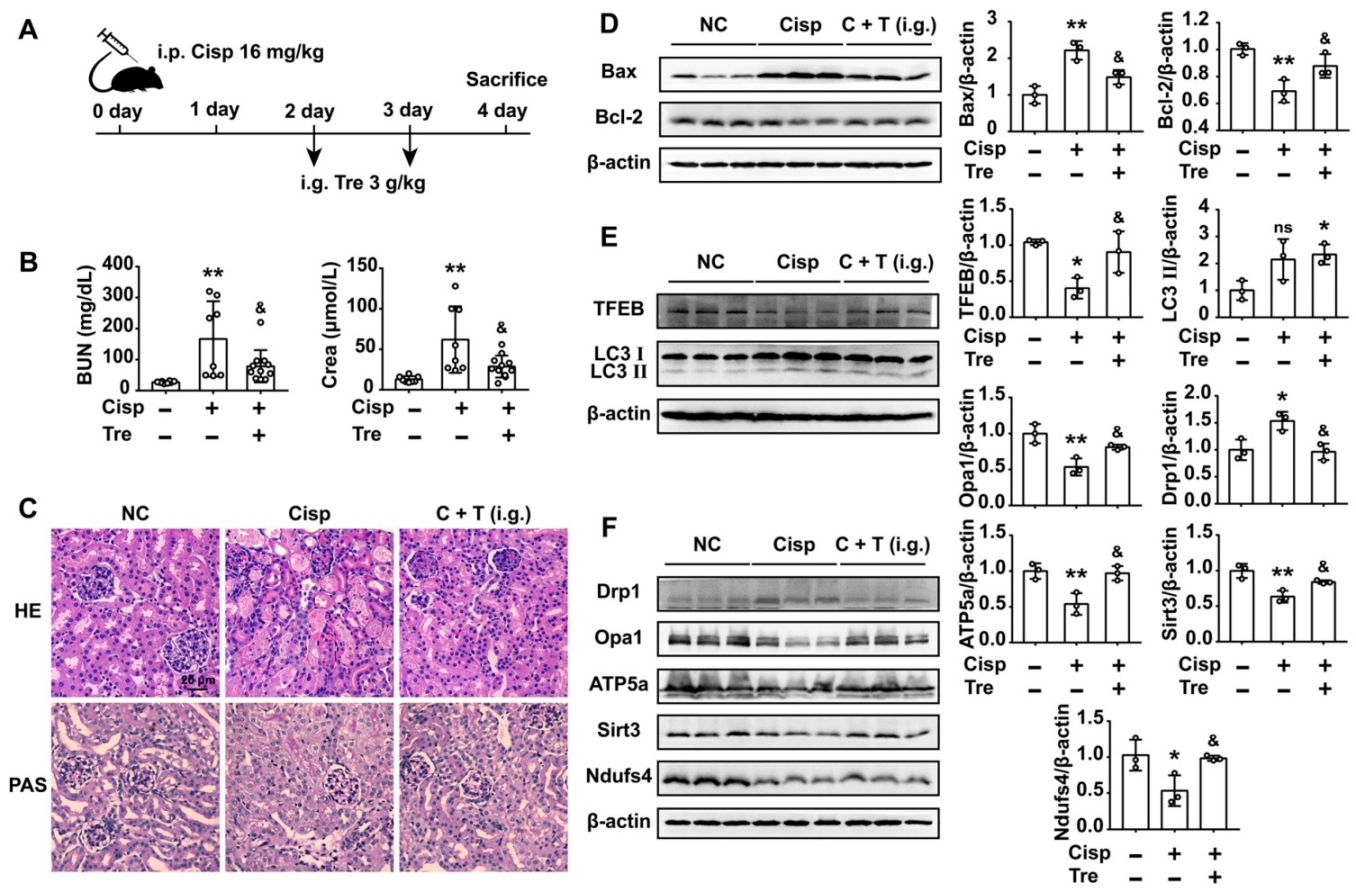
** Oral treatment with trehalose alleviates cisplatin-induced AKI.** (A) Mice were intraperitoneally injected with cisplatin, and trehalose was orally administered on the 2nd day after cisplatin injection. (B) Serum BUN and Crea levels were quantified in each group (n = 8 - 11 mice). (C) Representative images of HE- and PAS-stained kidney sections. (D - F) The expression of apoptosis-related proteins (Bax and Bcl-2), autophagy-related proteins (TFEB and LC3), and mitochondria-related proteins (Drp1, Opa1, ATP5a, Sirt3 and Ndufs4) was measured by western blotting (n = 3 mice). Data analyzed by one-way ANOVA with Tukey's test. * P < 0.05, ** P < 0.01 vs. Con; & P < 0.05, && P < 0.01 vs. Cisp. (NC, normal control; Cisp, cisplatin; Tre, trehalose; C + T, cisplatin + trehalose; i.g., intragastric; BUN, blood urea nitrogen; Crea, serum creatinine; HE, hematoxylin/eosin; PAS, periodic acid-Schiff).

We further investigated whether oral treatment with trehalose could alleviate kidney injury when renal function was impaired after cisplatin injection. In AKI mice, the BUN and Crea levels were increased on the 2nd day and continuously increased on the 4th day after cisplatin injection. Consistently, pathological damage to kidney tissue was clearly observed on the 2nd day after cisplatin injection (Figure S3C and D). To investigate the oral treatment of trehalose, trehalose was orally administered on the 2nd day after cisplatin injection (Figure 10A). The results showed that oral treatment with trehalose reduced BUN and Crea levels, inhibited apoptosis, and improved pathological structural damage (Figure 10B-D). Moreover, along with the promotion of autophagy in kidney tissue, mitochondrial function was improved after oral treatment with trehalose (Figure 10E and F).

## Discussion

In kidney diseases, trehalose has been found to ameliorate renal function in the polycystic kidney and Akt2 knockout-induced insulin resistance models 29. Thus, we hypothesized that trehalose might have potential as an effective drug to treat cisplatin nephrotoxicity. To our knowledge, the present study was the first to investigate the effects of trehalose on cisplatin-induced AKI.

Mitochondria undergo significant alterations during AKI 30, with functional and structural damage appearing earlier than the pathologic manifestations of kidney injury 31. Mitochondria are known as the main intracellular sites for ROS production 32. Under pathological conditions, mtROS can trigger oxidative stress, the inflammatory response and apoptosis, which ultimately results in kidney injury 9, 33. Consistent with previous studies, our study also found that mitochondrial function in cisplatin-induced HK2 cells and AKI mouse kidneys was impaired, as shown by mitochondrial fragmentation, the excessive accumulation of mtROS, and depolarization of the membrane potential. Thus, protection of the mitochondria might be a valuable therapeutic approach for the treatment of AKI.

Growing evidence supports that autophagy induction has been demonstrated in various experimental models of AKI, and promotion autophagy is protective 34-36. Moreover, inhibition of autophagy aggravated AKI, whereas activation of autophagy showed protective effects, suggesting the renal protective role of autophagy in AKI disease 37. Trehalose is regarded as an autophagy inducer in neurological studies 38. Moreover, the safety of trehalose is well recognized 39. Mitophagy, as a form of selective autophagy, targets impaired mitochondria for autophagic recognition and degradation, is important for maintaining mitochondrial quality 18, 40. Therefore, we investigated whether trehalose provided benefits to damaged mitochondria in AKI mice. Here, we revealed that trehalose induced autophagy both* in vitro* and *in vivo*, as shown by the increased expression of LC3 II. In terms of mitophagy, the expression levels of Pink1 and Parkin were increased after trehalose treatment. Moreover, the colocalization between LC3 and mitochondria was increased, indicating that the mitophagy process was activated. Due to the induction of mitophagy, mitochondrial function was improved in cisplatin-induced HK2 cells and AKI mice.

Autophagic responses to stress conditions depend on transcriptional regulation. TFEB is a known master transcriptional regulator of autophagy and can regulate lysosome biogenesis and promote autophagosome-lysosome activity by binding to the CLEAR element on a variety of autophagy and lysosomal genes to induce their expression 41, 42. Several studies have found that trehalose enhances the activity of autophagy by increasing activation of TFEB 28, 43, 44.

Moreover, Lynch/Parikh have found that suppressive effect of PGC1α on mitochondrial ROS (mtROS) induced by cisplatin was fully reversed by TFEB depletion. Moreover, the mitochondria damage, indicated by electron microscopy in cisplatin-treated iNephPGC1α mouse kidneys was exacerbated when the TFEB was knockdown, suggesting that TFEB-mediated mitophagy was pivotal for PGC1α-dependent renal stress resistance 18. Consistent with the observation, our results showed that after the trehalose treatment not only the suppression of mtROS, the ATP content and protein levels of Opa1, ATP5a, Ndufs4 in cisplatin-treated cells were elevated by TFEB-driven autophagy, while blocked in HK2 cells with TFEB knockdown. These findings indicate that trehalose upregulates autophagy and improves mitochondrial function by enhancing the nuclear translocation of TFEB. However, the mechanism involved in trehalose-mediated activation of TFEB is unclear.

Multiple studies have shown that the subcellular localization and activity of TFEB proteins are primarily regulated through phosphorylation. Phosphorylated TFEB is retained in the cytoplasm and subsequently translocates to the nucleus upon dephosphorylation 45, 46. Based on previous studies, transcription factor-mediated lysosome-to-nucleus signaling can be directly controlled by several signaling molecules involved in the mTORC1, ERK, and AKT pathways 45. As an inducer of autophagy, trehalose has been reported to induce autophagy via an mTOR-independent pathway 38. Previous studies have demonstrated that the nuclear translocation of TFEB mediated by trehalose is due to the inhibition of Akt-mediated phosphorylation of TFEB at S467, and pharmacological inhibition of Akt promotes TFEB nuclear translocation 47. Moreover, several experiments have confirmed that the Akt pathway is activated in cisplatin-induced HK2 cells and AKI mice 48, 49. Thus, we speculated that activation of TFEB mediated by trehalose in AKI mice may be dependent on the inhibition of Akt.

As an autophagy inducer, trehalose has been received the most attention in mammalian cells. However, some autophagy-independent pathway has been uncovered in recent studies. Lee et al have found that trehalose downregulates PARP-1 and PARP-2 expression, prevents lipopolysaccharide (LPS) and interferon gamma (INFγ) (a method widely used to induce iNOS and to promote neurotoxic conditions) induced oxidative injury and apoptosis in primary rat astrocyte and oligodendrocyte cells cultures 19. Moreover, it has been reported that trehalose not only promots autophagy but also functions as an activator of the p62-Keap1/Nrf2 pathway, leading to suppression of oxidative stress 27. Thus, the autophagy-independent pathway might also be involved in the nephroprotective effects of trehalose.

In our study, we demonstrated that trehalose treatment activated TFEB-mediated autophagy, alleviated the mitochondrial dysfunction and kidney injury in cisplatin-induced AKI mice.

## Supplementary Material

Supplementary figures.

## Figures and Tables

**Table 1:** Primers used for real-time PCR analysis

p62-F	CCGTCTACAGGTGAACTCCAGTCC
p62-R	AGCCAGCCGCCTTCATCAGAG
LC3b-F	CCGACTTATTCGAGAGCAGCATCC
LC3b-R	GTCCGTTCACCAACAGGAAGAAGG
Lamp1-F	CTCTGTGGACAAGTACAACGT
Lamp1-R	GTTGATGTTGAGAAGCCTTGTC
Ctsb-F	ATACTCAGAGGACAGGATCACT
Ctsb-R	ATCTTTTCCCAGTACTGATCGG
Becn1-F	GGAGCTGCCGTTATACTGTTCTGG
Becn1-R	TGCCTCCTGTGTCTTCAATCTTGC
Atg5-F	GATGGGATTGCAAAATGACAGA
Atg5-R	GAAAGGTCTTTCAGTCGTTGTC
TFEB-F	CAGCAGTCGCAGCATCAGAAGG
TFEB-R	TGTTGCCAGCGGAGGAGGAC
GAPDH-F	ACCACAGTCCATGCCATCAC
GAPDH-R	TCCACCACCCTGTTGCTGTA

## Abbreviations

AKI: acute kidney injury
BUN: blood urea nitrogen
CCK8: cell counting kit 8
Cisp: cisplatin
Cisp + Tre: cisplatin + trehalose
Con: control
Crea: serum creatinine
HCQ: hydroxychloroquine
Cisp + HCQ: cisplatin + hydroxychloroquine
mPRTCs: mouse primary tubule epithelial cells
NC: normal control
Rapa: rapamycin
ROS: reactive oxygen species
SDS-PAGE: sodium dodecyl sulfate-polyacrylamide gel electrophoresis
TFEB: transcription factor EB
Tre: trehalose
TUNEL: terminal deoxynucleotidyl transferase-mediated dUTP nick-end labeling staining

## References

[1] Gonsalez SR, Cortês AL, Silva RCD, Lowe J, Prieto MC, Silva Lara LD (2019). Acute kidney injury overview: From basic findings to new prevention and therapy strategies. Pharmacol Ther.

[2] Holditch SJ, Brown CN, Lombardi AM, Nguyen KN, Edelstein CL (2019). Recent Advances in Models, Mechanisms, Biomarkers, and Interventions in Cisplatin-Induced Acute Kidney Injury. Int J Mol Sci.

[3] Karasawa T, Steyger PS (2015). An integrated view of cisplatin-induced nephrotoxicity and ototoxicity. Toxicol Lett.

[4] Volarevic V, Markovic BS, Jankovic MG, Djokovic B, Jovicic N, Harrell CR (2019). Galectin 3 protects from cisplatin-induced acute kidney injury by promoting TLR-2-dependent activation of IDO1/Kynurenine pathway in renal DCs. Theranostics.

[5] Manohar S, Leung N (2018). Cisplatin nephrotoxicity: a review of the literature. J Nephrol.

[6] Volarevic V, Djokovic B, Jankovic MG, Harrell CR, Fellabaum C, Djonov V (2019). Molecular mechanisms of cisplatin-induced nephrotoxicity: a balance on the knife edge between renoprotection and tumor toxicity. J Biomed Sci.

[7] Oh CJ, Ha CM, Choi YK, Park S, Choe MS, Jeoung NH (2017). Pyruvate dehydrogenase kinase 4 deficiency attenuates cisplatin-induced acute kidney injury. Kidney Int.

[8] Guo Y, Ni J, Chen S, Bai M, Lin J, Ding G (2018). MicroRNA-709 Mediates Acute Tubular Injury through Effects on Mitochondrial Function. J Am Soc Nephrol.

[9] Ichinomiya M, Shimada A, Ohta N, Inouchi E, Ogihara K, Naya Y (2018). Demonstration of Mitochondrial Damage and Mitophagy in Cisplatin-Mediated Nephrotoxicity. Tohoku J Exp Med.

[10] Bajwa A, Rosin DL, Chroscicki P, Lee S, Dondeti K, Ye H (2015). Sphingosine 1-phosphate receptor-1 enhances mitochondrial function and reduces cisplatin-induced tubule injury. J Am Soc Nephrol.

[11] Jia P, Wu X, Pan T, Xu S, Hu J, Ding X (2019). Uncoupling protein 1 inhibits mitochondrial reactive oxygen species generation and alleviates acute kidney injury. EBioMedicine.

[12] Ravanan P, Srikumar IF, Talwar P (2017). Autophagy: The spotlight for cellular stress responses. Life Sci.

[13] Bian A, Shi M, Flores B, Gillings N, Li P, Yan SX (2017). Downregulation of autophagy is associated with severe ischemia-reperfusion-induced acute kidney injury in overexpressing C-reactive protein mice. PLoS One.

[14] Lin Q, Li S, Jiang N, Shao X, Zhang M, Jin H (2019). PINK1-parkin pathway of mitophagy protects against contrast-induced acute kidney injury via decreasing mitochondrial ROS and NLRP3 inflammasome activation. Redox Biol.

[15] Tang C, Han H, Yan M, Zhu S, Liu J, Liu Z (2018). PINK1-PRKN/PARK2 pathway of mitophagy is activated to protect against renal ischemia-reperfusion injury. Autophagy.

[16] Zhang Y, Wang L, Meng L, Cao G, Wu Y (2019). Sirtuin 6 overexpression relieves sepsis-induced acute kidney injury by promoting autophagy. Cell Cycle.

[17] Liu J, Livingston MJ, Dong G, Tang C, Su Y, Wu G (2018). Histone deacetylase inhibitors protect against cisplatin-induced acute kidney injury by activating autophagy in proximal tubular cells. Cell Death Dis.

[18] Lynch MR, Tran MT, Ralto KM, Zsengeller ZK, Raman V, Bhasin SS (2019). TFEB-driven lysosomal biogenesis is pivotal for PGC1α-dependent renal stress resistance. JCI Insight.

[19] Lee HJ, Yoon YS, Lee SJ (2018). Mechanism of neuroprotection by trehalose: controversy surrounding autophagy induction. Cell Death Dis.

[20] Tang Q, Zheng G, Feng Z, Chen Y, Lou Y, Wang C (2017). Trehalose ameliorates oxidative stress-mediated mitochondrial dysfunction and ER stress via selective autophagy stimulation and autophagic flux restoration in osteoarthritis development. Cell Death Dis.

[21] Evans T D, Jeong SJ, Zhang X, Sergin I, Razani B (2018). TFEB and trehalose drive the macrophage autophagy-lysosome system to protect against atherosclerosis. Autophagy.

[22] Yuan Y, Zhu L, Li L, Liu J, Chen Y, Cheng J (2019). S-Sulfhydration of SIRT3 by Hydrogen Sulfide Attenuates Mitochondrial Dysfunction in Cisplatin-Induced Acute Kidney Injury. Antioxid Redox Signal.

[23] Schafer JA, Watkins ML, Li L, Herter P, Haxelmans S, Schlatter E (1997). A simplified method for isolation of large numbers of defined nephron segments. Am J Physiol.

[24] Lee S, Huen S, Nishio H, Nishio S, Lee HK, Choi BS (2011). Distinct macrophage phenotypes contribute to kidney injury and repair. J Am Soc Nephrol.

[25] Klionsky DJ, Abdelmohsen K, Abe A, Abedin MJ, Abeliovich H, Acevedo Arozena A (2016). Guidelines for the use and interpretation of assays for monitoring autophagy (3rd edition). Autophagy.

[26] Ni HM, Bockus A, Wozniak AL, Jones K, Weinman S, Yin XM (2011). Dissecting the dynamic turnover of GFP-LC3 in the autolysosome. Autophagy.

[27] Mizunoe Y, Kobayashi M, Sudo Y, Watanabe S, Yasukawa H, Natori D (2018). Trehalose protects against oxidative stress by regulating the Keap1-Nrf2 and autophagy pathways. Redox Biol.

[28] Rusmini P, Cortese K, Crippa V, Cristofani R, Cicardi ME, Ferrari V (2019). Trehalose induces autophagy via lysosomal-mediated TFEB activation in models of motoneuron degeneration. Autophagy.

[29] Yu W, Zha W, Peng H, Wang Q, Zhang S, Ren J (2019). Trehalose Protects against Insulin Resistance-Induced Tissue Injury and Excessive Autophagy in Skeletal Muscles and Kidney. Curr Pharm Des.

[30] Wang J, Zhu P, Li R, Ren J, Zhang Y, Zhou H (2020). Bax inhibitor 1 preserves mitochondrial homeostasis in acute kidney injury through promoting mitochondrial retention of PHB2. Theranostics.

[31] Plotnikov EY, Pevzner IB, Zorova LD, Chernikov VP, Prusov AN, Kireev II (2019). Mitochondrial Damage and Mitochondria-Targeted Antioxidant Protection in LPS-Induced Acute Kidney Injury. Antioxidants.

[32] Yue C, Yang Y, Zhang C, Alfranca G, Cheng S, Ma L (2016). ROS-Responsive Mitochondria-Targeting Blended Nanoparticles: Chemo- and Photodynamic Synergistic Therapy for Lung Cancer with On-Demand Drug Release upon Irradiation with a Single Light Source. Theranostics.

[33] Duann P, Lin PH (2017). Mitochondria Damage and Kidney Disease. Adv Exp Med Biol.

[34] Mei S, Livingston M, Hao J, Li L, Mei C, Dong Z (2016). Autophagy is activated to protect against endotoxic acute kidney injury. Sci Rep.

[35] Livingston MJ, Dong Z (2014). Autophagy in acute kidney injury. Semin Nephrol.

[36] Xu X, Pan J, Li H, Li X, Fang F, Wu D (2019). Atg7 mediates renal tubular cell apoptosis in vancomycin nephrotoxicity through activation of PKC-delta. FASEB J.

[37] Kaushal GP, Shah SV (2016). Autophagy in acute kidney injury. Kidney international.

[38] Hosseinpour-Moghaddam K, Caraglia M, Sahebkar A (2018). Autophagy induction by trehalose: Molecular mechanisms and therapeutic impacts. J Cell Physiol.

[39] Ohtake S, Wang YJ (2011). Trehalose: Current Use and Future Applications. J Pharm Sci.

[40] Pickles S, Vigie P, Youle RJ (2018). Mitophagy and Quality Control Mechanisms in Mitochondrial Maintenance. Curr Biol.

[41] Di Malta C, Cinque L, Settembre C (2019). Transcriptional Regulation of Autophagy: Mechanisms and Diseases. Front Cell Dev Biol.

[42] Martini-Stoica H, Xu Y, Ballabio A, Zheng H (2016). The Autophagy-Lysosomal Pathway in Neurodegeneration: A TFEB Perspective. Trends Neurosci.

[43] Wang Y, Liu FT, Wang YX, Guan RY, Chen C, Li DK (2018). Autophagic Modulation by Trehalose Reduces Accumulation of TDP-43 in a Cell Model of Amyotrophic Lateral Sclerosis via TFEB Activation. Neurotox Res.

[44] Wu H, Chen H, Zheng Z, Li J, Ding J, Huang Z (2019). Trehalose promotes the survival of random-pattern skin flaps by TFEB mediated autophagy enhancement. Cell Death Dis.

[45] Puertollano R, Ferguson SM, Brugarolas J, Ballabio A (2018). The complex relationship between TFEB transcription factor phosphorylation and subcellular localization. EMBO J.

[46] Raben N, Puertollano R (2016). TFEB and TFE3: Linking Lysosomes to Cellular Adaptation to Stress. Annu Rev Cell Dev Biol.

[47] Palmieri M, Pal R, Nelvagal HR, Lotfi P, Stinnett GR, Seymour ML (2017). mTORC1-independent TFEB activation via Akt inhibition promotes cellular clearance in neurodegenerative storage diseases. Nat Commun.

[48] Tang J, Shi Y, Liu N, Xu L, Zang X, Li P (2018). Blockade of histone deacetylase 6 protects against cisplatin-induced acute kidney injury. Clin Sci (Lond).

[49] Potocnjak I, Domitrovic R (2016). Carvacrol attenuates acute kidney injury induced by cisplatin through suppression of ERK and PI3K/Akt activation. Food Chem Toxicol.

