# Supplementary material for: Activation of TFEB-mediated autophagy by trehalose attenuates mitochondrial dysfunction in cisplatin-induced acute kidney injury
Source: Theranostics. 2020 Apr 27;10(13):5829–44. doi: 10.7150/thno.44051 (PMC7255003; doi:10.7150/thno.44051)
Supplement: Supplementary file 1 — Supplementary figures. [file thnov10p5829s1.pdf]

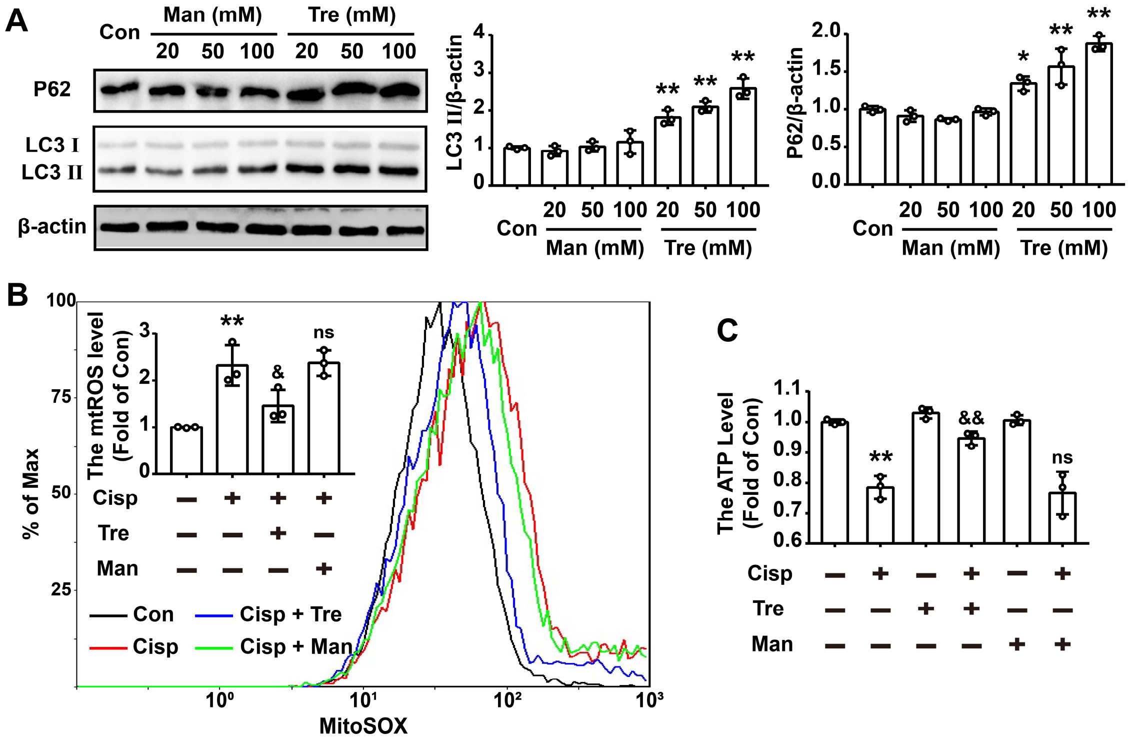

Figure S1 Trehalose induces autophagy and attenuates mitochondrial dysfunction in cisplatin-treated HK2 cells. Mannitol was used as osmotic control of trehalose. (A) The expression of the proteins P62 and LC3 was measured by western blotting in HK2 cells exposed to trehalose (20 mM, 50 mM, 100 mM) and mannitol (20 mM, 50 mM, 100 mM). (B) HK2 cells treated with cisplatin were incubated with trehalose or mannitol and mtROS were measured by incubation with MitoSOX. (C) ATP levels were quantified in HK2 cells, the ATP content was calculated as nmol/mg of protein, and the data are represented as the rate of control. (cisplatin, 5 μM; trehalose, 50 mM; mannitol, 50 mM). Data are shown as the means ± s.d. from three independent experiments and analyzed by one-way ANOVA with Tukey's test. * $P < 0.05$, ** $P < 0.01$ vs. Con; & $P < 0.05$, && $P < 0.01$ vs. Cisp. (Con, control; Tre, trehalose; Cisp, cisplatin; Man, mannitol; Cisp + Tre, cisplatin + trehalose; Cisp + Man, cisplatin + mannitol).

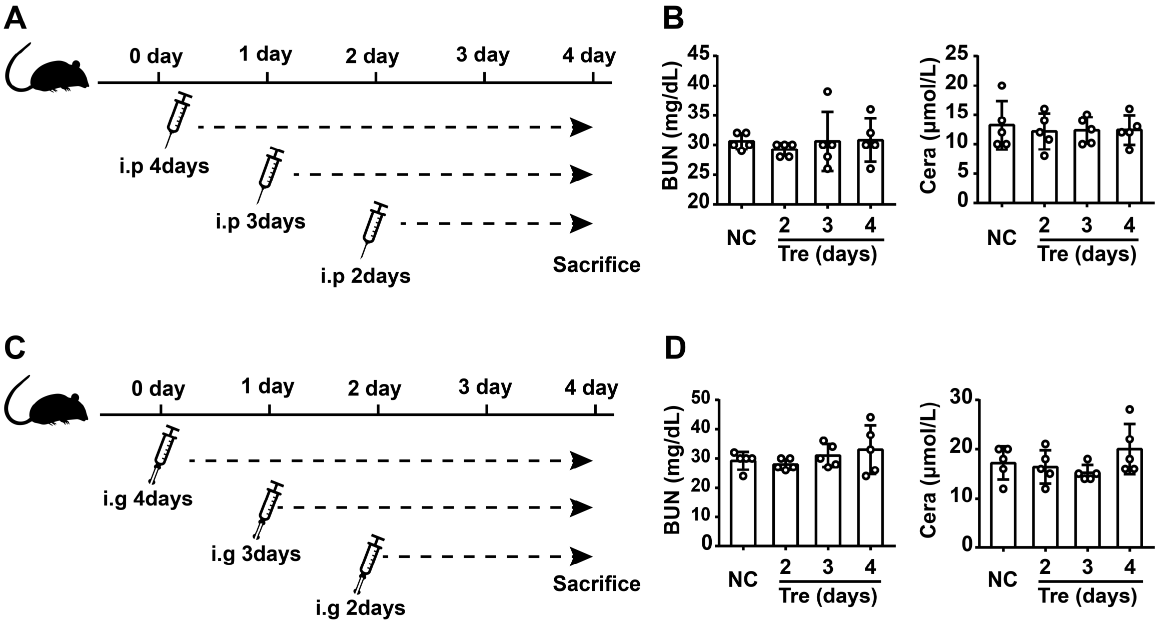

Figure S2 Administration of trehalose have no nephrotoxic effects to mice. (A and B) Mice were intraperitoneally injected with trehalose 2-4 days, and serum BUN and Crea were quantified in each group (n = 5 mice). (C and D) Mice were orally administrated with trehalose 2-4 days, and serum BUN and Crea were quantified in each group (n = 5 mice). Data are analyzed by one-way ANOVA with Tukey's test. (NC, normal control; Tre, trehalose; i.p., intraperitoneal; i.g., intragastric; BUN, blood urea nitrogen; Crea, serum creatinine).

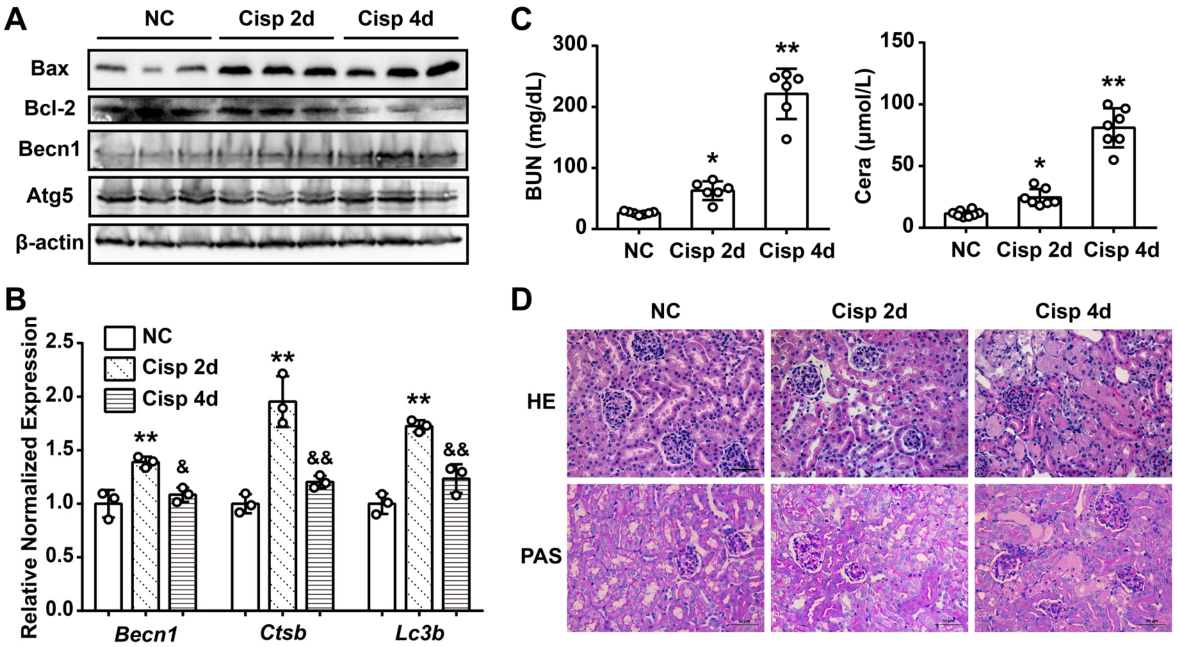

Figure S3 Cisplatin injection leads to kidney inury on the 2nd day in mice. (A) The expression of apoptosis-related proteins (Bax and Bcl-2), autophagy-related proteins (Becn1 and Atg5) was measured by western blotting. (B) The mRNA levels of several autophagy-lysosome markers were evaluated by real-time PCR (n = 3 mice). (C) Serum BUN and Crea levels were quantified in each group (n = 6 - 7 mice). (D) Representative images of HE- and PAS-stained kidney sections. Data analyzed by one-way ANOVA with Tukey's test. * $P < 0.05$, ** $P < 0.01$ vs. Con; $^{\&}P < 0.05$, $^{\&\&}P < 0.01$ vs. Cisp 2d. (NC, normal control; Cisp, cisplatin; BUN, blood urea nitrogen; Crea, serum creatinine; HE, hematoxylin/eosin; PAS, periodic acid-Schiff).

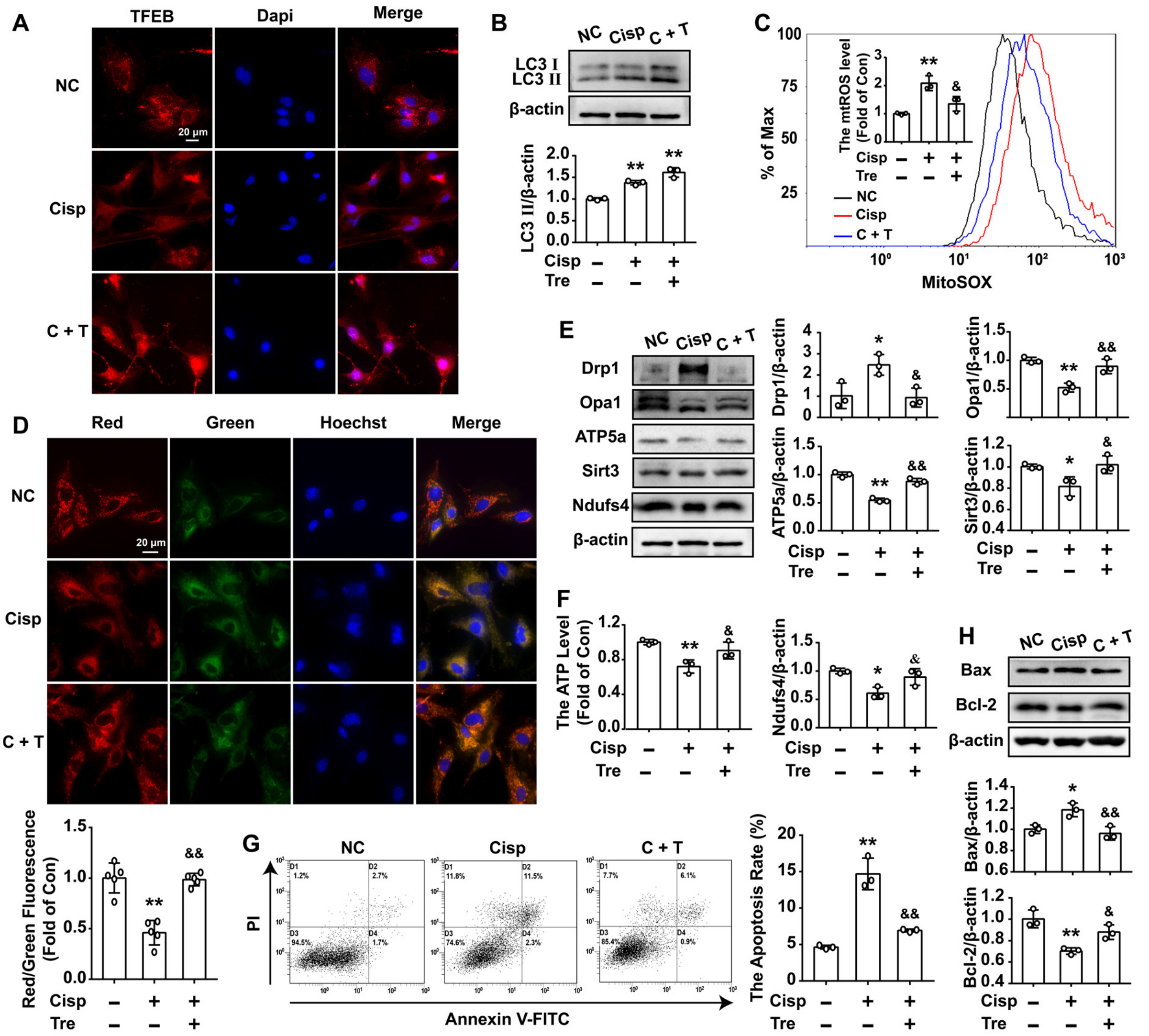

Figure S4 Trehalose alleviates mitochondrial dysfunction and apoptosis in the mouse primary tubule epithelial cells (mPRTCs) from cisplatin-induced AKI mice. (A) Representative immunofluorescence images of TFEB in mPRTCs. (B) Representative images of western blotting and quantitative analyses of LC3. (C) mtROS were measured by incubation with Mito-SOX. (D) The mitochondrial membrane potential measurement were detected with JC-1, and quantified by ImageJ (n = 5). (E) The expression of mitochondria-related proteins (Drp1, Opa1, ATP5a, Sirt3 and Ndufs4) was measured by western blotting. (F) ATP levels were assessed using an ATP assay kit. (G) The apoptosis of mPRTCs were determined by flow cytometry. (H) The expression of apoptosis-related proteins (Bax and Bcl-2) was measured by western blotting. Data are shown as the means ± s.d. from at least three independent experiments and analyzed by one-way ANOVA with Tukey's test. * $P < 0.05$, ** $P < 0.01$ vs. Con; $^{\&}P < 0.05$, $^{\&\&}P < 0.01$ vs. Cisp. (NC, normal control; Cisp, cisplatin; C + T, cisplatin + trehalose).
